# Neurophysiological markers of emotion regulation predict efficacy of entrepreneurship education

**DOI:** 10.1038/s41598-023-34148-1

**Published:** 2023-05-03

**Authors:** Pablo Egana-delSol, Xiaoxiao Sun, Paul Sajda

**Affiliations:** 1 School of Business, Universidad Adolfo Ibáñez, Santiago, Viña del Mar, Chile; 2 Millennium Nucleus on the Evolution of Work (MNEW), Santiago, Chile; 3 Department of Biomedical Engineering, Columbia University, New York, NY 10027 USA; 4 Department of Radiology, Columbia University Irving Medical Center, New York, NY 10032 USA; 5 Department of Electrical Engineering, Columbia University, New York, NY 10027 USA; 6 Data Science Institute, Columbia University, New York, NY 10027 USA

**Keywords:** Psychology, Human behaviour

## Abstract

Recent evidence shows that programs targeting the socio-emotional dimensions of entrepreneurship—e.g., resilience, personal initiative, and empathy—are more highly correlated with success along with key business metrics, such as sales and survival, than programs with a narrow, technical bent—e.g., accounting and finance. We argue that programs designed to foster socio-emotional skills are effective in improving entrepreneurship outcomes because they improve the students’ ability to regulate their emotions. They enhance the individuals’ disposition to make more measured, rational decisions. We test this hypothesis studying a randomized controlled trial (RCT, RCT ID: AEARCTR-0000916) of an entrepreneurship program in Chile. We combine administrative data, surveys, and neuro-psychological data from lab-in-the-field measurements. A key methodological contribution of this study is the use of the electroencephalogram (EEG) to quantify the impact of emotional responses. We find that the program has a positive and significant impact on educational outcomes and, in line with the findings of other studies in the literature, we find no impact on self-reported measures of socio-emotional skills (e.g., grit and locus of control) and creativity. Our novel insight comes from the finding that the program has a significant impact on neurophysiological markers, decreasing arousal (a proxy of alertness), valence (a proxy for withdrawal from or approachability to an event or stimuli), and neuro-psychological changes to negative stimuli.

## Introduction

The literature on behavioral economics claims that even minor emotional manipulations have a substantial impact on behaviors, decision-making, and economic outcomes^1–7^. Daniel Kahneman’s theory about human thinking holds that individuals possess two main systems: System 1, which is fast, instinctive, and emotional, and System 2, which is slow, deliberative, and logical^8^. In Kahneman’s view, emotion regulation drives people to behave based more on System 2 than on System 1, thus enabling them to identify and pursue more optimal outcomes for themselves and others. While definitions of the terms emotion regulation, responsiveness, and emotional self-regulation, may vary, we define emotion regulation as a mixture of cognitive and emotional processes that shape a mental state—i.e., a disposition to act by directing oneself to consciously affect one’s own emotional and actual response to given stimuli^9^.

Recent evidences show that programs targeting the socio-emotional dimensions of entrepreneurship are more highly correlated with success along key business metrics such as sales, survival, etc., than programs with a narrow, technical bent^10^. However, the literature on entrepreneurship education highlights the lack of well identified studies outlining the mechanisms behind the observed impact and, in particular, the lack of attention paid to the role that socio-emotional skills play in entrepreneurship education^11–14^. There is a vast literature in economics supporting the notion that socio-emotional skills—e.g., self-confidence, internal locus of control, and grit—are relevant for success in life along other dimensions such as salaries, occupational choice, etc^15–22^.

However, there is not a strong understanding of the mechanisms by which socio-emotional skills contribute to these measures of success. Testing for grit and locus of control, both important socio-emotional skills, has proven inconclusive^3,17,23–25^. In this paper we instead test the hypothesis that the enhancement of emotion regulation capacity is the main mechanism through which socio-emotional skills impact outcomes.

To conduct this research, we leverage a randomized control trial implemented by the non-governmental organization (NGO) Youth Entrepreneurship in the north of Chile, which provides exogenous variation in participation in entrepreneurship programs. This NGO worked with a sample of eight schools, four of which were randomly assigned to an intervention that consisted of semester-long weekly 90-min workshops during which the students participated in different activities designed to improve their creative, entrepreneurial, and socio-emotional skills.

To assess emotion regulation of the students in the program we used electroencephalography (EEG) recordings, employing methods borrowed from affective neuroscience. A major contribution of this paper is the use of neuroscientific techniques to quantify emotional regulation in large cohort, shifting the discussion from largely self-reported measures that form the basis of much of the literature on socio-emotional skills toward more objective brain-based measurements.

Specifically, we find a significant correlation between emotion regulation biomarkers and educational outcomes, which supports the fact that emotion regulation is a potential mechanism driving these findings. We also find that the program affects not only the mean of emotion biomarkers, but also the variance of their distribution. Overall, this evidence provides support to the claim that changes in students’ emotion regulation capabilities partially explain the observed impact on educational outcomes.

## Materials and methods

### The program to foster entrepreneurship

The NGO Youth Entrepreneurship, inspired by the model of David McClelland^26^, started a program in Chile to foster ten entrepreneurship and leadership skills through didactic weekly interventions. This model embraces the principles of “learning by failing, gaming, doing and rethinking”. McClelland^26^ tested more than 500 businesses, sports, religious and political leaders of the world with the intention of understanding which of their competencies meaningfully set them apart from the majority of people. His research postulated the presence of 30 socio-emotional skills that were relevant in this respect, 10 of which might be developed in the short term. These 10 skills are: (1) searching for opportunity and taking the initiative; (2) being persistent (gritty); (3) working in support networks; (4) searching for information; (5) taking calculated risks; (6) complying with work commitments; (7) systematically planning and monitoring; (8) being persuasive; (9) demanding efficiency and quality; (10) being self-confident. The program targeted young students, ages 16–18, in four high schools with a particular focus on self- confidence and creativity. The annual estimated budget of the program was approximately USD$100,000 for all four schools. The fixed cost (i.e., methodology design, experts, professionals, book design and videos) represented 64% of this figure, while the variable cost (i.e., books, workshops, monitoring, etc.) accounted for the rest. The estimated cost of the book was USD$24.

The program targeted young students ages 16–18 who were enrolled in the 12th grade of vocational high school (the last year of secondary education). The program was accompanied by the development of course materials, including a high-quality student hardcover textbook and a teacher textbook, the latter of which included class-by-class guidelines and videos for each activity to be performed. The program also included three pre-implementation training seminars and pilot/simulated training workshops that took place before the core curriculum was implemented in the teachers’ schools. Importantly, the program’s coaches worked together with schoolteachers to ensure the intervention was homogeneous across schools, and to generate local capabilities within each school.

The intervention was designed as follows. The classroom was transformed into a friendly game room. All school desks were put aside, and the chairs were arranged in a semi-circle to ensure that each student would sit the same distance from the center. The students were then taught to recognize their own strengths and weaknesses by acting through games and reflections/deliberations, working in teams under pressure, addressing complex tasks, and setting their own goals to be achieved through these tasks. Finally, a discussion about the learning objectives was conducted after each session.

### Sample selection

Together with the NGO that implemented the program, we invited eight Technical-professional (TPE) schools located in the IV Region of Chile. All invited schools wanted to participate in the program (see SI for more details).

The final sample of our neurophysiological data comprises an unbalanced panel with 296 valid EEG recordings that we use for the difference-in-difference modeling. In particular, we have 76 (64) and 104 (52) students in the treatment and control groups at baseline (follow-up), respectively. For the model to estimation of net effect of entrepreneurship program on the VA space, more students’ data are used (331 total EEG recordings, 85(68) and 110(68) students in the treatment and control groups at baseline (follow-up)), because we don’t need to eliminate students who miss personal information that is needed for the difference-in-difference model. Considering the time and complexity involved in collecting these neurophysiological data, especially in an out-of-the-lab setting, this was an extraordinary number of measures. Indeed, we collected more than 400 student EEG recordings. Filtering for artifacts and data quality gave us the final sample (see details in the SI). We also obtained administrative data from the Ministry of Education (MINEDUC) and the DEMRE, which is the institution that has the information on the SAT-like test that is a requisite to apply to universities in Chile. For MINEDUC we have 1,888 students and for DEMRE 1,296 students considering both treatment and control group. These samples are considerable larger because comprises all students in all eight schools considered that has public records on attendance and registration to the SAT-like test, respectively.

### Treatment

We define treatment as participation in the entrepreneurship program during the March–June academic semester in 2015. The intervention took place in the participants’ schools and consisted of weekly 90-min workshops during that semester. This study was approved by the institutional review board at Columbia University and written informed consent was obtained from all parents of the participants all participants and/or their legal guardians before the main experimental sessions. We also confirmed that all research was performed in accordance with relevant guidelines/regulations and the ethics of the project was binding with local standards in Chile.

### Randomization at the school level

Of the eight invited schools, we randomly chose four to participate in the program by simple random sampling without replacement. We collected baseline information from both the treatment and control schools in February 2015. All 12th grade high school students took part in the intervention in the treatment schools. We also considered 11th grade students attending both treatment and control schools as an additional comparison group. In the presence of positive spillover effects from the treated, considering this additional comparison group would only make our results just more conservative. All students attending 12th grade in the treated schools had to participate in the program as part of regular school obligations. This limits the concerns about selection bias due to non-random attrition. The mandatory feature also allows us to estimate average treatment effects, instead of intent to treat estimates.

### The team

The personnel for this study, excluding the administrators who helped with the internal logistics, comprised a small research team consisting of the authors of the study and a small number of research assistants who were trained by the authors in all the relevant areas of methodology, equipment, and theory.

### Portable laboratory

**Figure 1:**
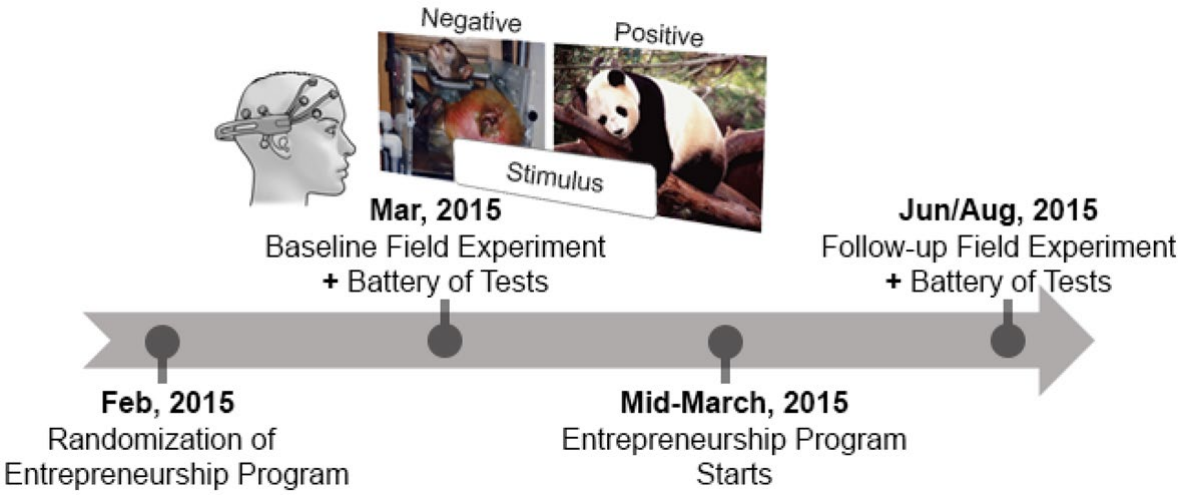
Experiment timeline and experiment paradigm. Four stages are included which are randomized selection, baseline field experiment, entrepreneurship program, and follow-up experiment, respectively. Entire experiment starts at February 2015 and ends in August 2015.

We built a portable laboratory containing seven workstations, each of which consisted of a portable EEG headset (Emotiv Epoc, Emotiv Co.) paired with a high-capacity laptop (Lenovo ThinkPad). This experimental setting, which had previously been piloted at Columbia University, enabled us to obtain a proxy measure of our students’ emotion regulation capabilities in a non-lab context (see Fig. 1 in the main text, Figs. S2 and S5 in the SI). The circumstances of data collection were homogeneous between treatment and control schools. Thus, it is reasonable to assume that there is no differential noise contamination to the data between treatment and control groups (e.g., external noise, time of the day, breakfast content, room lighting). We set up our portable labs in empty classrooms using white curtains to prevent direct external light in order to keep the contrast level as similar as possible across schools and students. Additionally, our field experiment was not conducted at full capacity (on average we have 6 students simultaneously doing the experiment). To mitigate any potential impact of proximity on the quality of the EEG recording, we instructed the students to use a device and seating area that was not close to other students who were also doing the experiment (i.e., spread the students out in the experiment room).

### Data collection

We conducted the baseline data collection in early March 2015 (see Fig. 1). We brought the portable neuro-scientific lab to all eight schools during this time. Then, the intervention (entrepreneurship program) started in mid-March 2015. The follow-up data collection happened when the first semester ended, or second semester started (June or August of the same year). Thus, treatment effects correspond to participation in the entrepreneurship program during one academic semester. We collected data on socio-emotional, creative, and cognitive abilities, as well as neurophysiological measurements. Most of the measurements took place in the morning during class. Since the field experiment was conducted in the same way across all schools, and the class schedules were very similar, we can assume that the data were collected around the same time in all schools, and thus similarly on average on the treatment and control groups. Below we explain each of these outcomes in detail. This study was reviewed and approved by the Institutional Review Board of Columbia University (IRB-AAAP2363) and written informed consent was obtained from all participants prior to enrollment. Permission was sought and given by the heads of all involved schools. This randomized control trial (RCT) was registered at the American Economic Association (AEA, RCT ID: AEARCTR-0000916).

### Self-reported socio-emotional skills measures

We collected self-report measures of the qualitative aspects of participants’ socio-emotional skills. We considered two main tests. First, the Grit Test, developed by Duckworth et al.^27^, measures individual’s perseverance to attain long term goals by asking to grade 12 statements in a likert-scale. Second, the Internal Locus of Control Test, a psychometric test developed by Julian Rotter^28^, that indicates the extent to which individuals believe that life events are under their control, using 20 statements that have to be graded using a likert-scale.

### Creative skills measures

In order to measure creativity, we employ an adaptation to the written version of the 1976 Torrance Test of Creative Thinking (TTCT)^29,30^, originally adapted to Chile by Pablo Egana-delSol^31^. We created an instrument to measure four dimensions of creativity: fluency, flexibility, originality, and elaboration (see more details of instrument on the SI).

### Cognitive skills measure: Raven’s progressive matrices

We used Progressive Matrices similar to those created by James Raven^32^ to measure fluid intelligence.

### Neurophysiological measurements and experiments

Over the past decades, emotion-detection research has employed a variety of physiological measurements and methods, including pupil dilation, heart rate, and skin conductance for arousal^33^, and voice and facial manifestations for valence^34–37^. However, many of these measurements can be consciously modified; thus, the signals they produce are not purely objective^36^. Therefore, we use EEG recordings, a more plausibly objective instrument to proxy emotion regulation abilities, which directly affect an individual’s disposition to act^9^, and which have been suggested as a relevant mechanism for other educational interventions targeting youths^25^. A number of authors have stated that EEG recordings can measure brain activity and predict emotional states and physiological responsiveness, improving both accuracy and objectiveness in respect to the aforementioned physiological measurements^34,36,38,39^.

To establish a more rigorous foundation for our research in the domain of objective measurement, we employ the James-Lange theory of valence and arousal.l^40^, which relies on neurophysiological measurements, to accurately capture and codify emotional data.

As illustrated by Fig. 1, our field work was primarily concerned with the collection of three sets of data: (1) a battery of psychometric tests; (2) resting emotional state (no stimuli) from EEG recordings of field experiment; (3) emotional responsiveness to both positive and negative stimuli from EEG recordings of field experiment.

The emotional state with no stimuli, also referred as “at resting state” measure, was constructed using EEG scalp recordings taken while the students watched a black cross in the center of a gray screen for a period of 30 s. We transformed the electrical recordings from the scalp into frequencies (i.e., cycles per second), and then interpreted the various frequencies, mainly alpha (8–12 Hz) and beta (12–30 Hz), and brain locations to estimate the students’ emotional states. By only extracting the alpha and beta frequencies, the influence of much noise is already significantly reduced, because the influence of eye movement or blinking artifacts is most dominant below 4 Hz, while heart related movements, i.e., Electrocardiogram (ECG), artifacts around 1.2 Hz, and facial muscle movements, i.e., electromyogram (EMG), artifacts above 30 Hz^41,42^ (see more detail in the SI). We estimate emotional arousal and valence indices at resting state using models from emotion-detection theory (see more detail in the Emotion-detection theory section of SI). As the Positive and Negative Affect Schedule (PANAS) is self-reported, it experiences issues such as test setting bias^43^ and reference bias^21^. An alternative can be the use of neurophysiological methods drawn from affective neuroscience^3,31^. In particular, arousal is constructed as a beta-alpha ratio on the pre-frontal cortex, while the left, relative to right (LFA), frontal cortical activity is used to build valence indices^34,41,42,44–48^.

After measuring emotion indices at resting state, we applied a psychometric test that includes the Grit scale, the locus of control scale, the raven-like progressive matrices, and the creativity test. We measured the participants’ emotional responsiveness immediately after the students had finished this test regimen. The experiment consisted of showing an alternating series of five positive and negative images in order to elicit emotional responses from each participant, which we illustrate in Fig. 1. These reactions were then converted to valence indices. We explain the technical details in the SI. Our study incorporates an estimate of arousal and three estimates of valence with regard to the nature or absence of stimuli (displaying stimuli within a virtual reality environment may be better suited to capture these differences in arousal and valence^49^ but was not a feasible option for us during the time when we conducted our field experiment). These were: (1) No stimuli, which captures student’s emotion responsiveness at resting state; (2) Positive Stimuli, which captures the responsiveness to positive stimuli net of the resting state measure; and (3) Negative Stimuli, which captures the response to negative stimuli net of the resting state measure. We normalize these indices relative to the mean and standard deviation of the control group. We then trim the indices at the 2.5 and 97.5 percentiles to avoid a substantial influence of the outliers of EEG measurement, as is common practice in literature^50,51^.

### Measured main educational outcomes

(1) Dropout Rates: In order to assess how and to what extent the program impacted the participants’ educational attainments, we assessed administrative data drawn from Ministry of Education (MINEDUC) to determine when and whether any students had withdrawn from their studies at any point in time. This process yielded a dichotomous variable, which took on the value “1” if a student had dropped out, and “0” if he/she had not. This administrative data was provided by the Ministry of Education under strict rules of privacy. We were able to collect information about all students enrolled in the eight schools that participated in the study, which substantially enlarged the usable sample.

(2) PSU Test: The PSU is similar to the SAT in the U.S.; it is optional for students who have completed secondary education but is required for those applying to university. When combined with the cumulative average of secondary education grades, the PSU represents the primary selection criterion for Chilean universities. In this regard, the PSU has a relative weight of around 70% in the final university application score. The PSU score is normalized to an average 500 points and a standard deviation of 100 points, with a fixed status ranging from 150 to 850 points. Apart from assessing whether or not the program would make students more or less likely to complete their compulsory secondary studies, we also wanted to gauge whether or not it would impact their desire to pursue further, post-secondary studies after completing high school. The willingness to consider post-secondary education is an indicator of better aspirations for their future. We therefore consider as a binary outcome of interest whether or not a student registered for the PSU. The data for this assessment, which were provided by the Department of Measurements (DEMRE) at the University of Chile, indicated which students had registered for the PSU.

### Statistical modeling

(1) Educational outcomes: We expected the entrepreneurship program to affect certain educational outcomes, such as registration to the university selection test (i.e., Prueba de Selección Universitaria, PSU) and high school dropout. Both outcomes could be thought as proxies of positive aspirations or motivation. Administrative data on educational outcomes are only accessible at the end of each academic year. We estimate a simple Probit model comparing treatment and control groups at the end of the year, which can be written as:

1 $$Y_{i,s}^{j} = \alpha^{j} + \beta^{j} \times Treat_{i} + \theta^{j} \times G_{i} + \gamma_{s}^{j} + \varepsilon_{i}^{j}$$

where indicates $Y_{i,s}^{j}$ educational outcome $j$ of student $i$ enrolled at school *s*. $j$ represents either PSU registration or an indicator for whether or not the student dropped out of high school, such that $Y_{i,s}^{j} = 1$ if a student is observed to either register for the PSU or drop out at the end of the year. *Treat*_*i*_ is a dichotomic variable equal to 1 if the student is enrolled in the program. *G*_*i*_ represent the gender of the student. In the dropout model we were able to include school fixed effects, represented by $\gamma_{s}^{j}$. These control variables were chosen based on the privacy restrictions imposed by DEMRE, which manages the PSU information, and MINEDUC, which provides the dropout data. Given these restrictions, we could only add gender and school information.

(2) Emotional regulation: A key contribution of this study is the use of a novel methodology, EEG recordings paired with the James-Lange theory of valence and arousal^40^, to measure neurophysiological markers associated with emotions. We use these measurements to examine whether the program induced changes in these measures, which we determine by employing the valence and arousal indices to gauge emotional reactions to stimuli, thereby testing for emotion regulation as a mechanism for observed changes in educational outcomes. First, the indices of resting state valence and arousal were measured as explained in *data collection* section. Second, we calculated the emotional responsiveness in the 2-D Valence-Arousal (VA) space^52^ (see Fig. S4 in the SI) to both positive and negative stimuli selected from the Geneva Affective Picture Database (GAPED).

We estimate a difference-in-difference model given by:

2 $$E_{i,s,t}^{j} = \alpha_{s} + \beta_{j} \times Treat_{i} + \gamma_{j} + Post_{t} + \delta_{j} \times Treat_{i} \times Post_{t} + \theta_{j} \times X_{i} + \varepsilon_{i,j,t}$$

where $E_{i,s,t}^{j}$ indicates the *j* emotion-related variable of individual *i* in school *s* at time *t*. The emotion-related variables indexed by *j* include Arousal (no stimuli), Valence (no stimuli), Valence (positive stimuli), and Valence (negative stimuli). The variables Valence (positive stimuli) and Valence (negative stimuli) measure the difference for each individual *i* at time *t* between student’s Valence (no stimuli) and the valence measured in student’s positive and negative stimuli scenarios, respectively. These indicators capture the emotional reaction net of their “resting state”, i.e., level of arousal and valence changes compared to no stimuli. We estimate each student “resting state” level of arousal and valence using EEG recordings taken while the students watched a black cross in the center of a gray screen for a period of 30 s. We consider a 5-s window in the middle of that time frame for our estimation. As before, *Treat*_*i*_ and *Post*_*t*_ are dichotomic variables representing treatment by the program and survey time—i.e., baseline or follow up—respectively. Finally, *X*_*i*_ indicates other controls at individual level, such as gender, while *α*_*s*_ represents the school level variables (i.e., fixed effects).

Additionally, we estimate the net effect of entrepreneurship on emotion responsiveness (i.e., valence and arousal) by measuring the difference of changes of emotion respect to negative/positive stimuli, which can be summarized by:

3 $$\Delta A_{s,g}^{t} = A_{s,g}^{t} - A_{no,g}^{t}$$

4 $$\Delta A_{s,g}^{\Delta t} = \Delta A_{s,g}^{t = 1} - \Delta A_{s,g}^{t = 0}$$

5 $$\Delta V_{s,g}^{t} = V_{s,g}^{t} - V_{no,g}^{t}$$

6 $$\Delta V_{s,g}^{\Delta t} = \Delta V_{s,g}^{t = 1} - \Delta V_{s,g}^{t = 0}$$

where $A_{s,g}^{t}$ represents the arousal changes of stimuli type *s* (*s* = 1 is positive or *s* = 2 is negative stimuli) at time *t* (*t* = 0 is baseline or *t* = 1 is follow-up) for different groups *g* (*g* = 0 is student without program, or *g* = 1 is student with program), $\Delta A_{s,g}^{t}$ measures the difference at time *t* between student’s emotion response measured in student’s different stimuli scenarios *s* (either positive or negative) compared to the resting state ($A_{no,g}^{t}$, no stimuli condition). $\Delta A_{s,g}^{\Delta t}$ measures the difference between time *t* ($\Delta t = 1 - 0$, i.e., follow-up subtracts baseline) of student’s $\Delta A_{s,g}^{t}$. For $V_{s,g}^{t}$, $\Delta V_{s,g}^{t}$ and $\Delta V_{s,g}^{\Delta t}$, everything is the same except that *V* standards for the measurement of valence. These indicators capture the emotional reaction net of their “resting state”, i.e., no stimuli ($A_{no,g}^{t}$ and $V_{no,g}^{t}$), between two time periods, which compares the effect of the entrepreneurship program.

With the difference-in-difference model and measurement of net effect, we can control for all time-invariant characteristics within and between groups. By subtracting the average differences between groups before the program started and only comparing the difference after the program, we can account for any pre-existing differences between the groups. This methodology also helps to control for any other unobserved variables that may affect both groups in a similar way.

(3) Socio-emotional Skills, Creativity, and Cognitive Skills: The entrepreneurship program was expected to positively impact the socio-emotional skills of its participants. Using conventional self-reported tests, we measured grit (perseverance) and locus of control. Additionally, the program was expected to impact creative and innovative skills, which are a mixture of both cognitive and socio-emotional skills. Note that the literature demonstrating best practices for measuring creativity remains scarce, particularly in the context of educational program evaluations^31^. Similar to the previous model, we control for differences before the program started—i.e., at baseline—between treatment and control groups.

7 $$Y_{i,t}^{j} = \alpha_{s} + \beta_{j} \times Treat_{i} + \gamma_{j} \times Post_{t} + \delta_{j} \times Treat_{i} \times Post_{t} + \theta_{j} \times X_{i} + \varepsilon_{j,i,t}$$

In this specification, $Y_{i,t}^{j}$ indicates the test score for exam *j* of individual *i* at time *t*. The index *j* represents one of creativity, grit, or internal locus of control. *Treat*_*i*_ is a dichotomic variable equal to 1 if the school received the program, and *Post*_*t*_ is a dichotomic variable equal to 0 at baseline (pre-treatment) or equal to 1 at follow-up (post-treatment). Finally, *X*_*i*_ indicates school-level and 12th grade dichotomic variables.

In order to achieve the highest possible statistical power in these estimations, we used both non-treated 12th grade (*N* = 61) and 11th grade (*N* = 95) students in the control group in all specifications. We include grade indicators to control for unobserved differences in cohorts that might be correlated with the entrepreneurship intervention.

## Results

### Educational outcomes

Only 42% of students in Technical and Professional Education (TPE) schools in Chile continue on to higher education, compared with 66% of the students in Scientific Humanistic High School Education (SHE) programs^53^. Additionally, only 39% of those TPE students who do continue to higher education complete their degrees. One potential reason for this discrepancy is that TPE students lack the ability to complete higher education. This explanation is reflected in their achievements across several dimensions. After controlling for demographics and institutional characteristics, TPE schools under-perform SHE schools in grades, access to higher education, and degree completion rates, among others^54^.

We report estimates of educational outcomes (see Eq. (1)) in Table 1. Since information about registration for the university selection test comes from administrative records, we can track this outcome for all students in treatment and control groups, not only the ones that participated in the neuropsychological measurements. Therefore, we estimate Eq. (1) for both the full sample of students targeted by the entrepreneurship program and for the subset for whom we have information about other outcomes. In Columns (1) and (2) of Table 1, we show that in both samples, there is a statistically significant increase in PSU registration as a result of enrollment in the program.

**Table 1:** Educational outcomes.

Variables	(1)	(2)	(3)
	PSU^a^	PSU^a^	Dropout^b^
Treatment^c^	0.130***	0.309***	− 0.068*
	(0.028)	(0.056)	(0.037)
Gender			− 0.002
			(0.010)

Sample	DEMRE^e^	EEG SAMPLE	MINEDUC^f^
Observations	1296	294	1888
School FE^d^	No	No	Yes

Robust standard errors in parentheses: *** indicates *p* < 0.01, ** indicates *p* < 0.05, * indicates *p* < 0.1.

^a^“PSU” is a dichotomic variable equal to 1 if a student registered for the PSU, and 0 otherwise.

^b^“Dropout” is a dichotomic variable equal to 1 if a student dropped out of high school and 0 otherwise. Only students in their last year of secondary school are considered in the analysis.

^c^The marginal effects of the Probit model are reported for Column (3) only.

^d^Control variables were only available for registered students. Due to privacy concerns, DEMRE was unable to provide information on which school each student attended. This means we were not able to include school fixed effects for the DEMRE sample. Nevertheless, DEMRE did allow us to include an indicator for one unique school in the sample which was public but run by a Catholic congregation rather than the local municipality.

^e^DEMRE is the official administrator of the PSU test.

^f^MINEDUC is the Ministry of Education of Chile. Due to privacy restrictions, we cannot report dropout rates just for the RCT sample.

In Column (3) of Table 1 we show that the program leads to a six percentage points decline in the probability of dropping out of high school. Here, we can only present results using the full administrative sample from MINEDUC. The magnitude of the decline is substantial due to the fact that dropout rates are relatively high in Chile, especially among students from vulnerable socio-economic backgrounds who are over-represented in technical schools^54^. Moreover, descriptive analyses of similar programs previously implemented by Youth Entrepreneurship show positive impacts of such programs in terms of lowering dropout rates among participants relative to historical rates^55^. As this study is concerned with identifying the mechanisms through which these outcomes occur, such as the program’s effects on emotion regulation or socio-emotional skills, any further discussion of the impacts on these educational outcomes lies beyond its scope.

### Emotion regulation

In the difference-in-difference model of emotional regulation (see Eq. (2)), we hypothesize that ${\delta }_{j}<0$ for arousal and valence in the no stimuli condition, and for valence in the negative stimuli scenarios, while ${\delta }_{j}=0$ for valence in the positive stimuli situations. The expected values for ${\delta }_{j}$ are consistent with the asymmetry found in the literature where emotionally laden stimuli that are negative have a significant effect on decision-making and behaviors, while positive stimuli seem to have no effect^2,56^. We also expect $\beta =\gamma =0$. We find the effects to be consistent with our hypothesis. However, because this methodology is novel, and there is not yet a comparable literature to which we can compare our findings.

**Figure 2:**
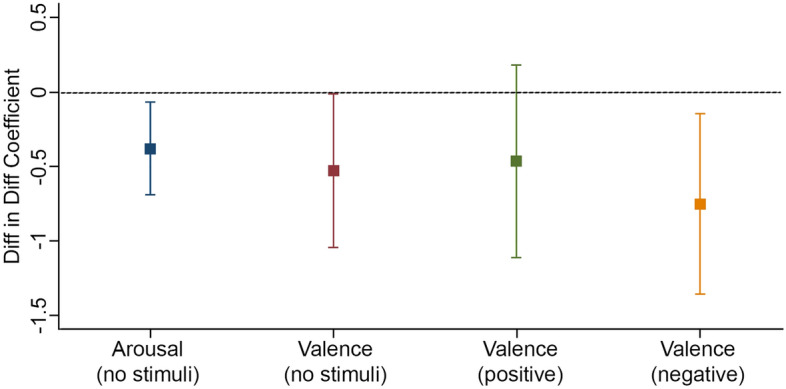
Impact on Emotional Responsiveness. The result of *δ*_*j*_ for different emotion-related variables *j* are shown. Bars represent the model estimation and 95% CI. Except Valence (positive), all other three variables have *δ* significantly (*p* < 0.05) smaller than 0 and the impact on responsiveness to negative stimuli Valence (negative) is the strongest.

Figure 2 shows the results of estimating Eq. (2). We find that program participants present lower resting state arousal and valence indices than the control group. The measured impacts are of 0.13 standard deviations (hereafter $\sigma$) and 0.44 $\sigma$ for resting state arousal and valence, respectively. Moreover, we show in Fig. 2 that the intervention has a significant impact on responsiveness to negative emotionally laden stimuli, of approximately 0.47 $\sigma$, which is near the upper bound of similar interventions^57,58^. In other words, when faced with negative stimuli (e.g., pictures of mistreated animals or human rights violations) after treatment, the students’ neurophysiological responsiveness changed significantly less compared with the emotional reactions in the control group. This might be interpreted as an increase in the resilience trait among participants^59^.

**Figure 3:**
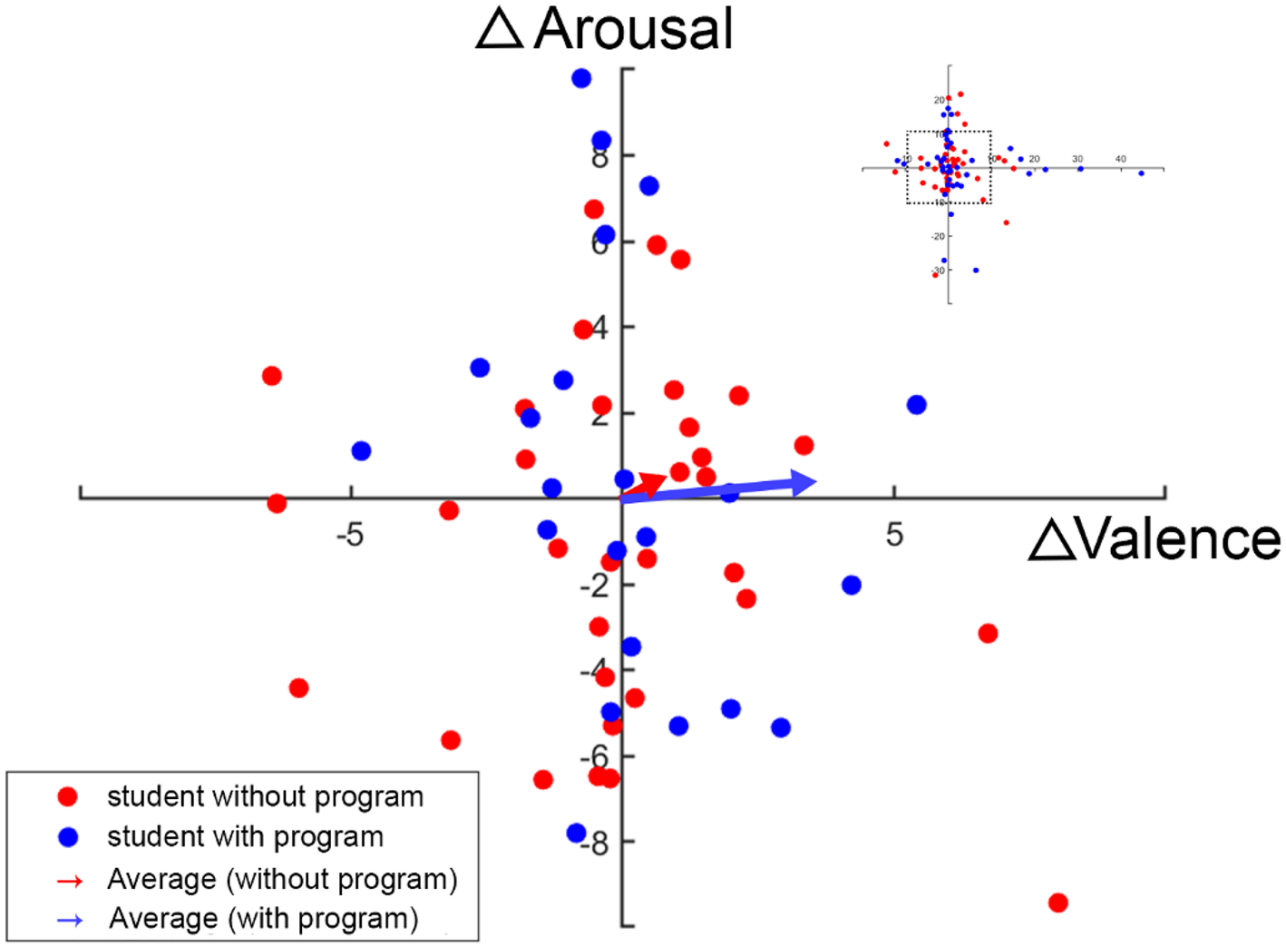
EEG biomarker changes in 2-D Valence-Arousal (VA) space for positive stimuli. EEG biomarker changes are between baseline (*t* = 0, *N*_0_ = 68) and follow-up (*t* = 1, *N*_1_ = 68) experiment (see Eqs. (4) to (6)). The complete scatter plot, one point per student, with its original scale is shown in the upper right corner. The scatter plot in the center is a zoomed-in version with [− 10, 10] as x/y-axis limit (x-axis: $\Delta Valance$; y-axis: $\Delta Arousal$). Blue dots represent students who participated in the entrepreneurship program (students with program, *g* = 1) and red dots represent students who do not participant the program (students without program, *g* = 0). Colored vectors show the average of each group in the VA space (e.g., the blue vector is determined by the value of both $\mu \left( {\Delta V_{s = 1,g = 1}^{\Delta t} } \right)$ and $\mu \left( {\Delta A_{s = 1,g = 1}^{\Delta t} } \right)$ . For students with program, the mean and standard error of changes in valence with respect to positive stimuli (i.e., $\Delta V_{s = 1,g = 1}^{\Delta t}$) is 3.51 ± 3.46 with a t-test showing that $\Delta V_{s = 1,g = 1}^{\Delta t}$ is significantly different from $\Delta Valance = 0$ with a 95% confidence interval ($\Delta V_{s = 1,g = 1}^{\Delta t} > 0$, *p* = 0.0495). However, for students without program, the mean and standard error of changes in valence with respect to positive stimuli (i.e., $\Delta V_{s = 1,g = 0}^{\Delta t}$) is 0.72 ± 1.64 and the t-test shows that $\Delta V_{s = 1,g = 0}^{\Delta t}$ is not significantly different from $\Delta Valance = 0$ within a 95% confidence interval. For changes in arousal domain ($\Delta Arousal$), there is no significant difference between $\Delta A_{s = 1,g = 0}^{\Delta t}$ (0.47 ± 2.52) and $\Delta A_{s = 1,g = 1}^{\Delta t}$ (0.36 ± 3.10) and neither are significantly different from $\Delta Arousal = 0$.

**Figure 4:**
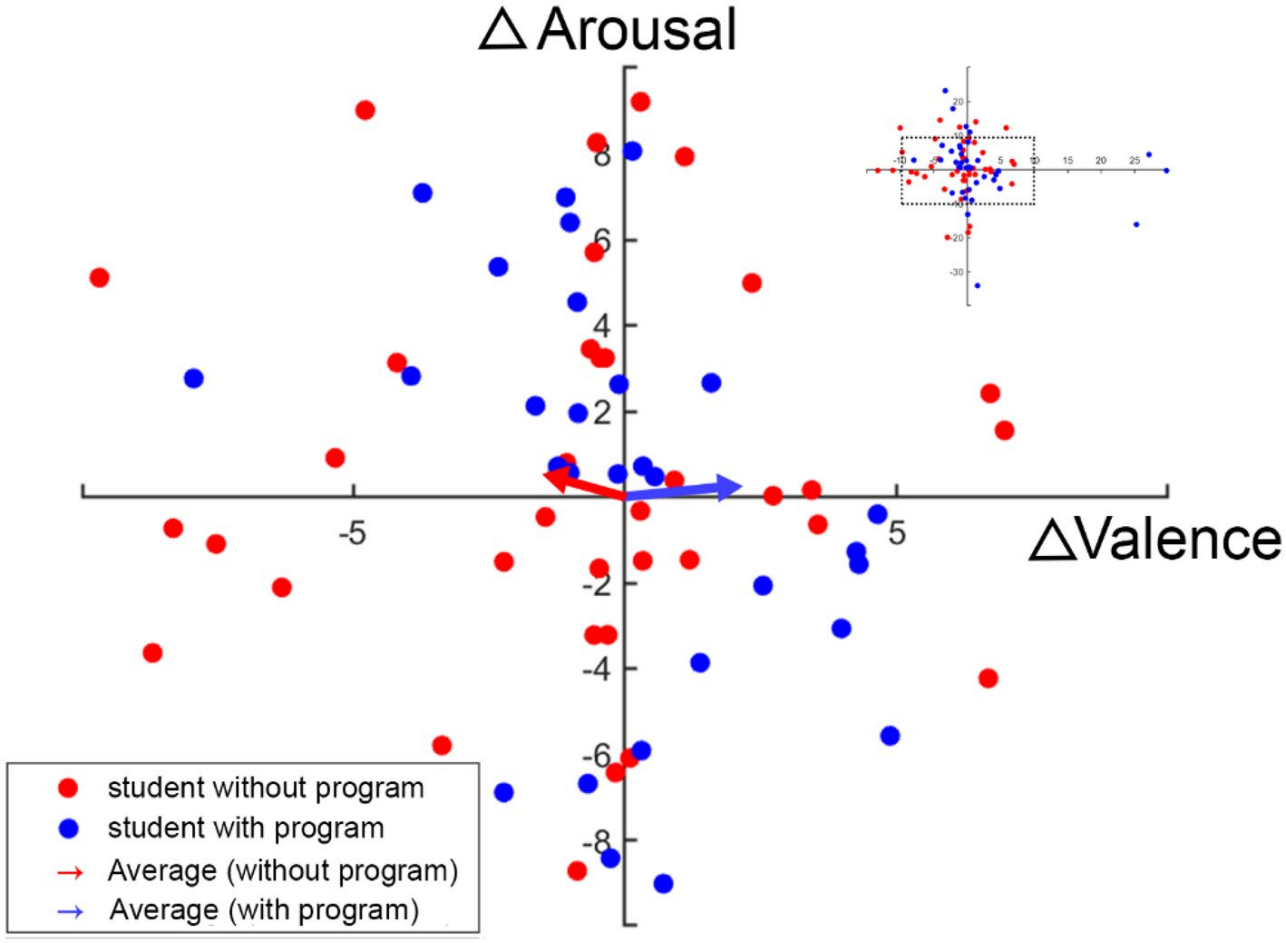
EEG biomarker changes in 2D Valence-Arousal (VA) space for negative stimuli. EEG biomarker changes are between baseline (*t* = 0, *N*_0_ = 68) and follow-up (*t* = 1, *N*_1_ = 68) experiment (see Eqs. (4) to (6)). Plot is structured the same as above, except that the comparison is for negative stimuli. For students with program (*g* = 1), the mean and standard error of changes in valence with respect to negative stimuli (i.e., $\Delta V_{s = 2,g = 1}^{\Delta t}$) is $2.08\pm 2.58$ with a t-test showing that $\Delta V_{s = 2,g = 1}^{\Delta t}$ is not significantly different from $\Delta Valance$ equals to 0 within a 95% confidence interval. However, for student without program (*g* = 0), the mean and standard error of changes in valence respect with respect to positive stimuli (i.e., $\Delta V_{s = 2,g = 0}^{\Delta t}$) is $-1.44\pm 1.37$ and the t-test shows that $\Delta V_{s = 2,g = 0}^{\Delta t}$ is significantly different from $\Delta Valance$ equals to 0 within 95% confidence interval ($\Delta V_{s = 2,g = 0}^{\Delta t} < 0$, *p* = 0.0414). With the t-test between two groups, results shows that there is significant difference between them. For changes in arousal domain ($\Delta Arousal$), there is no significant difference between $\Delta {\rm A}_{s = 2,g = 0}^{\Delta t}$ ($0.58\pm 2.22$) and $\Delta {\rm A}_{s = 2,g = 1}^{\Delta t}$ ($0.18\pm 3.10$) and neither of them is significantly different from $\Delta Arousal = 0$.

For the net effect of entrepreneurship on emotion responsiveness, we hypothesize that different student group would have different arousal and valence changes in different stimuli type due to the entrepreneurship program. Therefore, we evaluate whether those differences in different conditions (e.g., $\left( {\Delta V_{s = 1,g = 0}^{\Delta t} ,\Delta A_{s = 1,g = 0}^{\Delta t} } \right)$, $\left( {\Delta V_{s = 1,g = 1}^{\Delta t} ,\Delta A_{s = 1,g = 1}^{\Delta t} } \right)$ are significantly different from the origin of valence-arousal (VA) space. Figure 3 shows the results of these calculations based on Eqs. (4) and (6) with respect to positive stimuli in the 2-D VA space. We find that program participants present higher valence changes than the control group with positive stimuli ($\mu \left( {\Delta V_{s = 1,g = 1}^{\Delta t} } \right) > \mu \left( {\Delta V_{s = 1,g = 0}^{\Delta t} } \right)$, see Fig. 3), although this difference is non-significant (unpaired *t*-test: $\Delta V_{s = 1,g = 1}^{\Delta t} > \Delta V_{s = 1,g = 0}^{\Delta t}$, *p* = 0.1265). No significant difference in arousal indices between groups was found with respect to positive stimuli. In Fig. 4, however, it shows that the intervention has a significant impact on responsiveness to negative emotionally laden stimuli ($\mu \left( {\Delta V_{s = 2,g = 1}^{\Delta t} } \right) > \mu \left( {\Delta V_{s = 2,g = 0}^{\Delta t} } \right)$, unpaired t-test: $\Delta V_{s = 2,g = 1}^{\Delta t} > \Delta V_{s = 2,g = 0}^{\Delta t}$, *p* = 0.0134), which has similar impact in order of magnitude relative to similar interventions^57,58^ and it is consistent with the result reported from Eq. (2).

### Socio-emotional skills, creativity, and cognitive skills

**Figure 5:**
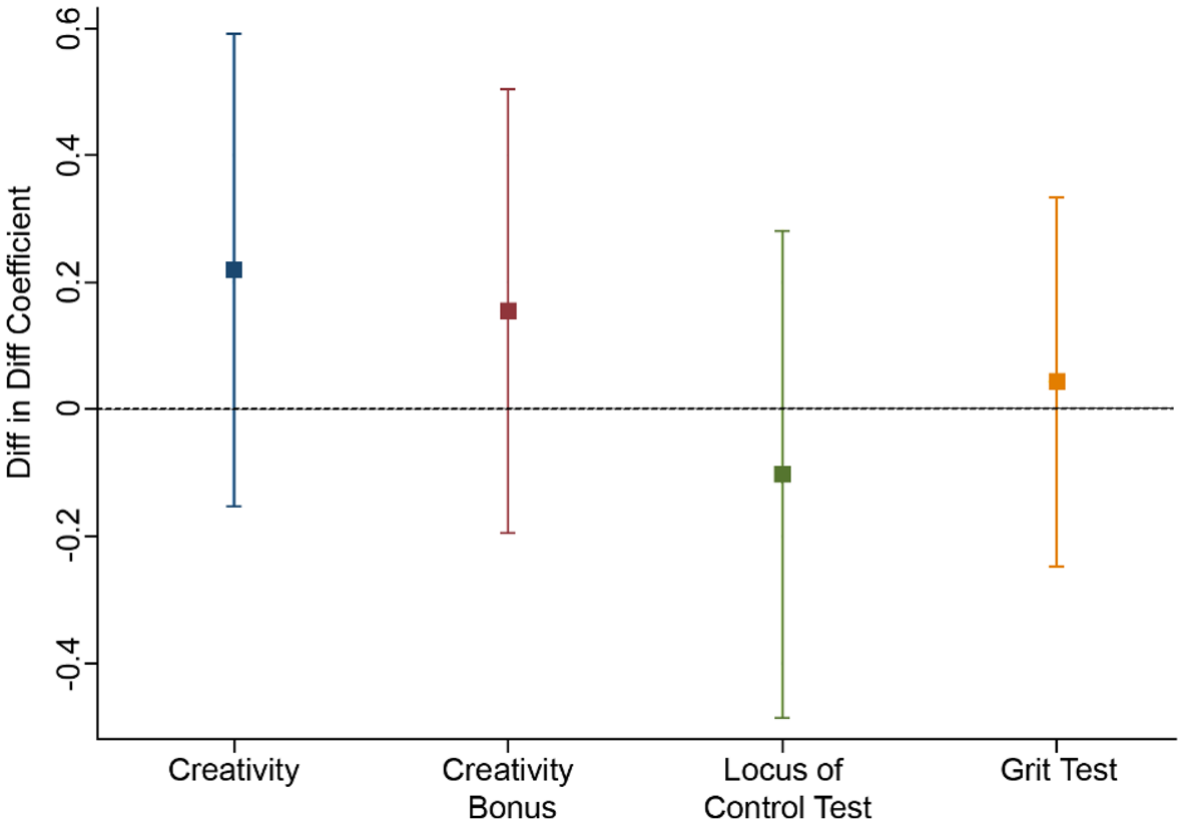
Impact on Creativity and Socio-emotional Skills. The result of ${\delta }_{j}$ for different self-reported test *j* are shown. Bars represent the model estimation and 95% CI. *δ* of all four tests are not significantly (*p* < 0.05) different from 0.

According to the hypothesis, socio-emotional skills are also expected to be affected by this entrepreneurship program. Here, we present the results from estimating those skills (see Eq. (7)) in Fig. 5. We report the main parameter of interest, ${\delta }_{j}$ with their 95% confidence interval. These parameters identify the average treatment effect by taking into account both the natural evolution of the outcome across time and the selection into the treatment group. Figure 5 shows that program participation did not impact the grit or locus of control scales in a statistically significant way, which is contrary to the program’s expected positive impact.

Given the results in Fig. 5, we interpret that the impact noted on the grit scale was likely driven by a difference in the treatment provided to 12th-grade participants, rather than by the program itself. The locus of control scale exhibited no significant change. However, the coefficient for this measure was negative, which indicates a positive correlation between internal locus of control and educational and labor outcomes^60^. Furthermore, the program’s estimated impact on both the creativity indices was not significant. Estimations were made using either a simple average of the three dimensions of creativity—flexibility, fluidity and originality—or a principal component analysis (PCA) index of creativity exhibited similar results in terms of magnitude, sign and significance.

We report the results of the entrepreneurship program’s impact on cognitive skills, as measured by Raven’s test of progressive matrices in the SI. As the entrepreneurship program was not designed to affect cognitive skills, this test was only incorporated in order to rule out any change in cognitive skills as a mechanism of observed impact on educational outcomes. We performed this test only in the follow up measurements, which is why we only have 116 observations. The lack of significant result suggests that changes in cognitive skills is the not channel through which the entrepreneurship program affects educational outcomes.

## Discussion

### Program participation impacts on emotion regulation instead of cognitive or other socio-emotional skills

In our research, we do not find strong evidence for changes in cognitive skills as a result of participation in the entrepreneurship program. This result likely stems from the program’s focus on socio-emotional skills as well as the age of the targeted population. This result is consistent with related work, such as Almlund et al.^15^ and reports from the Organisation for Economic Cooperation and Development (OECD)^61^, who find that cognitive skills explain only a small fraction of the variance found in labor market outcomes. We also find that participation in the entrepreneurship program does not impact self-reported measures of socio-emotional skills that were specifically targeted by the program’s curriculum, such as internal locus of control and grit. This result is also unsurprising, and commonly found in the literature^17,18,21–24^. These null results were expected and documented before running the RCT^62^.

Although the discussion of the role played by socio-emotional skills in the production function of entrepreneurs is still open, numerous researchers have highlighted these skills as a key component of entrepreneurial activity and the need to incorporate emotional dimensions in the analysis^11,13,63,64^.

We find that the program had a significant impact on the students’ emotional state, as indicated by a decrease in arousal and valence indices at resting state, based on EEG recordings. We also estimate significant declines in the participants’ emotional responsiveness to negative stimuli that we interpret as an increase in the ability to regulate emotions among participants. Since the focus is on learning from failure or adverse outcomes, it is unsurprising to find no significant response to positive stimuli. Considering the statistical significance of these results, the magnitude of the impact (i.e., from 0.13 $\sigma$ to 0.47 $\sigma$), and the methodological contribution to the evaluation of social programs, we next offer a discussion of how to interpret our findings in light of the literature on behavioral economics and affective neuroscience.

### Feeling, fast and slow

The link between emotions and cognition has been debated and explored by scholars for centuries^65^. Even today, prominent neuroscientists continue to explore the critical role played by emotions in cognition, perception, attention, and memory^9,66–68^. Furthermore, scientists who combine neuroscience, behavioral science, and economics also support the notion that emotions have a strong influence on economic behaviors and decision-making, as well as on labor market performance (e.g., occupational choices, salaries, entrepreneurship, etc.)^3,5,69,70^. For example, emotions experienced while making a decision—i.e., choice-option-elicited emotions—are at the base of traditional economic interpretations of utility as emotional carriers of value. Positive emotions increase value and elicit approach, whereas negative emotions decrease value and result in avoidance^3^. Moreover, emotions unrelated to the judgment or decision at hand, referred as incidental emotions, have also been shown to influence choices^3^.

Following Kahneman’s ideas, we argue that emotion regulation drives people to behave based more on System 2 than on System 1. System 2, given its more deliberate focus, enables individuals to identify and pursue better outcomes for themselves and others. We argue that the program studied here changes emotion regulation capabilities among participants, essentially pushing them toward behavior closer to System 2, which in turn affects their educational outcomes. In this section, we focus on how to interpret the results on neurophysiological measures and how the program affects emotion regulation. We also discuss why the program affects students‚ “disposition to act” and the economic relevance of our findings when compared to similar programs. We argue that the program affects emotion indices via a reappraisal strategy triggered by learning by failure. This reappraisal strategy, in turn, helps individuals develop a sense of resilience.

The behavioral economics literature provides evidence showing that even minor mood manipulations have a substantial impact on outcomes and behavior^2^. Schaffer et al. suggest an “approach/withdrawal” model to investigate left relative to right frontal cortex EEG asymmetry (LFA) regarding emotional states^71^. Since then, the literature on psychology and neurophysiology has pointed out that frontal EEG asymmetry is associated with different emotional and psychological states in addition to valence^42,44,72^. In fact, the “approach/withdrawal” model invites us to think of an additional interpretation of our results. A decrease in frontal EEG asymmetry (i.e., valence index) is consistent with a relative increase in withdrawal behaviors. This is mainly due to the fact that students would like to approach positive stimuli and avoid negative stimuli. Since the entrepreneurship program aims at instilling a more resilient, even-tempered attitude toward failure, this is a plausible interpretation of our results.

The asymmetry in the impacts of positive and negative emotional states is consistent with other findings in the literature. In Querengasser and Schindler’s work^73^, the authors referred to Randolph M. Nesse^74^, who argued that “Emotional states not only motivate action, they are also goals that we seek to achieve. Most human thought, plans, and actions are intended to induce positive emotions or to avoid negative emotions”. From this evolutionary point of view, a successful injunction of negative emotion would be more relevant for participants’ behaviors because negative emotions suggest a situation that should be altered, while positive emotions indicate situations that should be maintained^73,74^.

At first glance, the negative impact on emotional disposition would seem counterintuitive given that the program was designed to improve socio-emotional skills. For instance, changing the attitude toward failure would also change education and labor market decisions, such as efforts put into tasks, occupational choices, entrepreneurship, and the pursuit of creative and original work, among many others. This interpretation is consistent with the impact observed on educational outcome.

A core aspect of the program’s methodology was to generate situations in which students had to face failure and reappraise their emotions in such a context. Lerner et al.^7^ review the recent findings for “Solutions that Seek to Minimize the Emotional Response.” The results of these studies point to four dimensions of emotional responses: Time delay, Suppression, Reappraisal, and the “Dual-emotion solution”. Reappraisal consists of re-framing the meaning of those stimuli that lead to an emotional response and has consistently emerged as a superior strategy for dissipating the emotional response^7,75^. Specifically, reappraisal includes behaviors such as reminding oneself that “it’s just a test” after receiving a sub-optimal grade in an exam, adopting a mindset similar to that of a nurse or medical professional to minimize the emotional impact of viewing someone’s injury, or viewing a job layoff as an opportunity to pursue long-forgotten dreams^75^. In contrast to suppression, reappraisal not only reduces self-reported negative feelings in response to negative events, but there is substantial evidence supporting the notion that reappraisal also mitigates the physiological and neural responses to those events^69,76,77^. Additionally, regulating emotion by means of reappraisal-focused strategies that encourage taking a different perspective has been shown to reduce loss aversion in decision-making^78^. This result has been observed both in choices and in the relative arousal responses to actual loss and gain outcomes. Finally, there is evidence that reappraisal-focused strategies lead to higher measures of resilience among participants, which is consistent with the hypothesis that the program impacts this skill^59^. In addition, and according to Casey et al., the role played by positive and negative stimuli may be even more salient among adolescents^79^. In fact, the greater emotional responsiveness and sensitivity typical of this time of life may play a role in the higher incidence of the onset of affective disorders and addictive behaviors that often occurs during these years. Thus, policies aimed at increasing an individual’s ability to regulate his/her emotions may be even more meaningful when targeted toward adolescents.

Finally, it is hard to translate the estimated results into meaningful behavioral explanations. All emotional indices built with the EEG recordings were standardized with respect to the mean and standard deviation of the control group. Since Mathersul et al.^80^ reported that handedness does not appear to have any significant effect on the detection of emotion signatures in EEG, we did not explicitly measure the participants’ handedness in this experiment. However, considering the handedness and brain lateralization would be helpful to reduce statistical sensitivity, as some fields of cognitive neuroscience may not exclude left-handers a priori but may nevertheless do so a posteriori^81^. Furthermore, given the context of our RCT, the best that we can offer here is a comparison in terms of the effect size as a fraction of the standard deviation of the variables with respect to similar evaluations found in the literature for educational settings. In this regard, the impacts of 0.13 $\sigma$, 0.44 $\sigma$, and 0.47 $\sigma$ on arousal at resting state, valence at resting state, and responsiveness to negative stimuli, respectively, are near the upper bound of similar interventions^57,58^.

There are a number of papers that consider valence or left relative to right (LFA) cortical activity, to build behavioral indices of emotions or motivation. For instance, Hughes et al. examined the relationship between valence and effort expenditure for reward, a behavioral index of approach motivation^82^. They find that students with greater resting state valence were willing to expend greater effort in the pursuit of tasks with larger rewards, particularly when reward delivery was less likely.

## Conclusion

Our work has analyzed the impact of an entrepreneurship program implemented in vocational schools in Chile. This entrepreneurship program was designed for individuals to learn from their failures. We find that emotion regulation is the main channel through which the program impacts educational outcomes. A novel feature of our study is the use of EEG recordings as a measure of socio-emotional skills, grounding our discussion of emotion regulation in objective measurements rather than self-reported results, as in earlier studies. To be clear, we do not claim that EEG recordings (or plotting them along arousal and valence indices) record or “read” emotions; rather, these tools serve as useful proxies to measure changes in skills associated with emotion regulation. This promising use of EEG recordings complements recent work that uses cortisol as a physiological measure of psychological well-being in impact evaluations^56^.

We argue that training programs designed to foster socio-emotional skills affect the participants‚ capacity for emotion regulation, which is a unique form of socio-emotional skill. We test our hypothesis in two ways. First, we leverage a randomized controlled trial of an entrepreneurship program designed to foster socio-emotional skills among youth to get exogenous variation in program participation. Second, we use emotion-detection methods drawn from affective neuroscience to obtain unbiased measures of emotion regulation. We also document the limitations of self-reported measures of emotional states.

We find that the program has a significant impact on educational outcomes as measured by dropout rates and registration to the college entrance exam PSU. We do not find a significant impact on self-reported socio-emotional skills measures, which is consistent with the findings of previous related studies^21,24,25,83^. We do find, however, that the program impacts participants’ emotion regulation ability, as measured by our use of EEG recordings and plotting this data along arousal and valence indices.

On one hand, we find significant impacts on emotional state at resting state—i.e., a no stimuli condition. Specifically, we estimate a decrease in both arousal and emotional valence indices among participants. After analyzing the results, we establish that the impacts on emotion regulation are likely due to the reappraisal-focused strategies espoused by the program’s rethinking and redoing methodology. Indeed, these strategies have been identified as the most effective for avoiding emotional bias on decision-making^7^. On the other hand, program participation also significantly reduces individuals’ emotionally related neuro-physiological reactions to negative stimuli compared to students who have not taken the program. We do not find a significant difference between the two groups in response to positive stimuli. This asymmetry in the reactions to emotionally-laden stimuli is consistent with the asymmetric impact on emotional responsiveness found in the literature^2^. This might be interpreted as an increase in the resilience trait among participants^59^.

As mentioned before, the magnitude of our findings are close to the upper bound of similar educational interventions^57,58^. Moreover, these results provide a quantitative support to recent suggestive evidence based on qualitative analyses pointing out that programs targeting socio-emotional skills might affect the ability to young people to slow down and re-think about their response to a stimulus^25^. Finally, the emotion regulation indices correlate with our main educational outcome, which supports the hypothesis that emotion regulation is the mechanism behind our findings.

From a methodological perspective, the experimental paradigm adopted for this study provides an alternative to the approaches most frequently employed in the literature: self-reported measures, latent factor models, and the use of revealed behaviors as proxies. The methodology proposed here enabled us to measure emotional disposition and responsiveness by means of EEG recordings. The use of these recordings provides a solution that overcomes the biases related to self-reporting, and the question of whether programs such as the entrepreneurship one studied here impact any unobservable characteristics besides those that were initially targeted. Finally, the use of this methodology is at the cost of having a smaller sample size given all the logistics and technicality involved in the use of EEG devices.

## Supplementary Information

Supplementary Information.

## Data Availability

The datasets generated during and/or analyzed during the current study are available in the Harvard Dataverse repository, https://doi.org/10.7910/DVN/KE5SYF. This dataset has been anonymized (i.e., without RUT and names, etc.) before uploaded to the Harvard Dataverse.
