# Supplementary material for: Neurophysiological markers of emotion regulation predict efficacy of entrepreneurship education
Source: Sci Rep. 2023 May 3;13:7206. doi: 10.1038/s41598-023-34148-1 (PMC10156692; doi:10.1038/s41598-023-34148-1)
Supplement: Supplementary file 1 — Supplementary Information. [file 41598_2023_34148_MOESM1_ESM.docx]

**Supplementary Information for**

Neurophysiological Markers of Emotion Regulation Predict Efficacy of Entrepreneurship Education

Pablo Egana-delSol, Xiaoxiao Sun, Paul Sajda

Pablo Egana-delSol

Email: pegana@mit.edu

Supplementary Information Text

**Literature related to emotions in economics.** We argue that by being forced to confront failure not just once but multiple times, participants develop valuable emotional-regulation skills, affecting emotional indices via a reappraisal strategy that helps students develop resilience. Reappraisal strategy might be defined as reframing the meaning of those stimulus that lead to an emotional response; for example, reminding oneself that it’s just a test after receiving a sub-optimal exam grade, adopting a mindset similar to that of a laid-off employee who views his or her unemployment as an opportunity to pursue long-forgotten dreams^1^. In contrast to suppression, reappraisal not only reduces self-reported negative feelings in response to negative events, but there is substantial evidence supporting the notion that reappraisal also mitigates the physiological and neural responses to those events ^2-4^. Regulating emotion by means of reappraisal-focused strategies that encourage taking a different perspective has been shown to reduce loss aversion in decision-making ^5^. In developing the ability to form a reappraisal strategy, students move closer to Kahnemans System 2 thinking, which in turn affects their educational outcomes. Reviewing recent findings for “Solutions that Seek to Minimize the Emotional Response”, Lerner et al. ^6^ identifies four dimensions of emotional responses: Time delay, Suppression, Reappraisal, and the “Dual-emotion solution^[[1]](#footnote-1)^”. Finally, there is evidence that reappraisal-focused strategies lead to higher measures of resilience among participants, which is consistent with the hypothesis that the program impacts this skill ^7^. In addition, according to Casey et al., the role played by positive and negative stimuli may be even more salient among adolescents ^8^. In fact, the greater emotional responsiveness and sensitivity typical of this time of life may play a role in the higher incidence of the onset of affective disorders and addictive behaviors that often occurs during these years. Thus, policies aimed at increasing an individual’s ability to regulate his/her emotions may be even more meaningful when targeted toward adolescents.

We would like to state that 1) we are not claiming to be able to “measure” emotions, and 2) we explain the science behind this proxying more fully in **the Why doesn’t self-reporting work to proxy emotions** and **Emotion-detection theory** part. This contribution is two-fold, in the sense that at best, EEG recordings may prove a useful means of proxying emotions and emotional regulation; at worst, our methods and results may help expand the uses of these neurophysiological measures in interesting and fruitful ways.

Along with utilizing EEG recordings to quantify emotion regulation, we employ the widely accepted and well-studied neurophysiological “arousal” and “valence” markers to quantify the results obtained from those EEG recordings. These markers are associated with “positive” and “negative” emotions in the emotion-detection literature. Again, while such a decision may seem speculative, we have made sure to build on well-established evidence from the neuroscience literature when building emotion-related indices and correlating them with behavioral or performance outcomes ^9-18^ (see **From neuro-physiological indices to measuring emotional regulation** section for more details on this evidence).

The use of self-reported measures of emotional states is common in the literature, but they are subject to biases ranging from the test setting ^19^ and reference group bias ^20,21^, to the examinees’ emotional and motivational states ^22,23^, among others ^24,25^. Moreover, the intervention itself might affect emotional dimensions, which adds the additional challenge of disentangling treatment effects on particular skills from effects on emotions ^23,26^. Given that socio-emotional skills are one of the main outcomes of interest in our study, we decided to avoid self-reported measures of emotional regulation to avoid priming on the expected impact^[[2]](#footnote-2)^. Instead, we use emotion-detection methods drawn from affective neuroscience, which we believe are the best available techniques to objectively proxy individuals’ emotional states.

**The Role of Emotions on Decisions, Learning and Entrepreneurship.** The link between emotions and cognition has been debated and explored by scholars for centuries ^27^. Even today, prominent neuroscientists continue to explore the critical role played by emotions in cognition, perception, attention, and memory ^27-30^.

Furthermore, scientists who combine neuroscience, behavioral science, and economics also support the notion that emotions have a strong influence on economic behaviors and decision-making, as well as on labor market performance (e.g., occupational choices, salaries, entrepreneurship, etc.) ^2,31-33^. For example, emotions experienced while making a decision -- i.e., choice-option–elicited emotions -- are at the base of traditional economic interpretations of utility as emotional carriers of value. Positive emotions increase value and elicit approach, whereas negative emotions decrease value and result in avoidance ^31^. Moreover, emotions unrelated to the judgment or decision at hand, referred as incidental emotions, have also been shown to influence choices ^31^.

Given the role played by emotions in cognition, it is unsurprising that they also have implications on behaviors. For instance, Durlak et al. ^34^ conducted a meta-analysis on programs designed to develop emotional intelligence in educational settings. They found that the program positively impacted the participants’ targeted socio-emotional skills and attitudes about self, others, and school. They also argued that programs of this nature lead to higher levels of pro-social behavior, fewer instances of problems stemming from poor conduct or from the inability to internalize issues, and improved performance on academic achievement tests and grade point average. Similarly, a preschool and early primary school program designed to foster emotional intelligence demonstrated a positive impact on classroom behaviors and on executive functions, defined as higher level cognitive skills that include inhibitory control, working memory, and cognitive flexibility ^24,35^.

Moreover, there is a vast literature supporting the notion that socio-emotional skills -- e.g., self-confidence, internal locus of control, and grit -- are relevant for success in life ^20,25,36,37^. However, these studies highlight the positive impacts on economic outcomes (educational, entrepreneurial, or labor market-related), but find negligible or negative impacts on self-reported measures of socio-emotional skills. These results conflict with both intuition and conventional wisdom in the field and have thus been the subject of much scrutiny. Specifically, this conundrum emerges in recent research related to active labor market policies (ALMP) ^38-40^, educational programs ^20,41^, and entrepreneurship education programs ^42-45^.

This trend of behavioral outcomes resulting from encouraging emotional regulation extends into the majority of mainstream experiments and programs designed to impact educational or labor market outcomes. In many of these cases, it may even be argued that, whether or not this was the intention of the program directors, emotional regulation is the primary dimension targeted by the intervention. Heller et al. found suggestive evidence that programs such as Chicago’s “Become a Man (BAM)” influence participants to take more time when considering how to best formulate a response to an emotional stimulus; these students also demonstrated a lower propensity than their control-group peers to commit crime or drop out of school ^41^. By contrast, Haushofer and Fehr argued that the sub-optimal economic behaviors that make it difficult to escape poverty may be encouraged by the psychological consequences of poverty itself ^46^. According to evidence drawn from the literature, these psychological consequences include stress and negative emotional states, which, in turn, may lead to short-sighted and risk-averse decision-making, possibly by limiting attention spans and causing those so afflicted to favor habitual, instinctual behaviors at the expense of those that might result from long-term planning and goal-orientation. Together, these relationships may constitute a feedback loop that contributes to the perpetuation of poverty, which has been referred to as the “psychological poverty trap”.

Understanding how the emotional states of individuals influence their willingness and capacity to engage in productive behaviors has thus become a key goal for administrators and policymakers. Given the aforementioned presence, within modern development mandates, of entrepreneurship as a force for job creation, innovation, and self-employment, this question becomes even more salient when training programs are considered. Improving the understanding of emotions on other dimensions of entrepreneurship -- e.g., on the creation on new businesses, patents of innovations -- has recently been highlighted as one of the most important areas in which to pursue future research ^47^. These sentiments highlight the need for entrepreneurship training programs designed to feature emotional components in order to ensure that these efforts are effective. This has led to a plethora of studies seeking to understand which fundamental, socio-emotional attributes contribute most meaningfully to the development of entrepreneurial talent.

Although the discussion of the role played by socio-emotional skills in the production function of entrepreneurs is still open, numerous researchers have highlighted these skills as a key component of entrepreneurial activity ^42,48-50^.

**Why doesn’t self-reporting work to proxy emotions.** A number of different approximations are used to measure socio-emotional skills. A recent survey featured the use of behaviors -- drug use, registered behavior at school, etc. -- as well as both peer- and self-reported measures ^25^. For instance, Heckman et al. used risky behaviors -- i.e., the consumption of marijuana during adolescence -- to predict later outcomes in the labor market ^51^. Hirschi and Gottfredson argued that objective behavioral measures might be preferred to self-reports, as filling out a survey requires some level of self-control ^52^. Additionally, the proclivity to engage in risky and irresponsible behaviors, such as theft or the consumption of marijuana, during adolescence may be rooted in contextual factors that are not rooted in the self (upbringing, influence of peer groups, etc.).

Using measures similar to those applied to administrative data, Jackson investigated the effect wielded by teachers on their students' cognitive and socio-emotional skills ^53^. In particular, the author proxied cognitive skills by means of achievement test scores and socio-emotional skills by means of absences, suspensions, grades, and grade progression. These measures of character are commonly available from school administrative records and predict adult outcomes nearly as accurately as measures of cognitive ability. Some scholars criticize this approach, arguing that it is tautological to use measures of behavior to predict other behavior, even though future forecasts of behavior are extrapolated from past instances of it ^25^.

For example, a meta-analysis conducted by Pratt and Cullen found that behavioral measures are at least as good at predicting crime as those based on self-reported taxonomies ^54^. Moreover, administrative -- or third party -- data, which is frequently name-checked, is not always accessible in the context of a field experiment. Another option would be to consider external evaluations of the subjects. However, behaviors reported by peers, teachers, or parents could be biased, especially if those reporting know that these behaviors will be used to make decisions that could affect them. For instance, a school might like to assess the degree of grit among their students by asking teachers to evaluate students along this dimension. The teachers, in turn, might infer that the results of such an assessment would influence their own evaluations, and thus be incentivized to over-rate particular skills in their students.

Moreover, as mentioned previously, self-reporting may be influenced by test setting ^19^, reference bias ^20,21^, and by the examinees' emotional and motivational states at the time of the evaluation ^22,23,26^. In the context of well-designed assessments, it is plausible to argue that the impact of the test setting would be comparable for each examinee. By contrast, reference bias is not only one of the most common caveats for self-reported measures of socio-emotional skills, but it also skews results in ways that are highly personal and unpredictable. For example, in the Grit Scale statement “I am a hard worker”, the assessment ranges from “Very much like me (1)” to “Not like me at all (5)” ^55^. Thus, a subject's reference group will determine his/her self-perception -- a student from a high achieving school might consider him/herself relatively lazy, whereas a comparable one from a more standard school may consider him/herself especially fastidious^[[3]](#footnote-3)^ ^20^. Finally, as discussed in Egana-delSol's work ^26^, self-reported measures of socio-emotional skills are also affected by emotional/motivational states.

In summary, self-reported tests are sub-optimal instruments for the measurement of socio-emotional skills. In response to this assertion, we considered an alternative method to assess such skills, with a particular emphasis on emotional regulation. In the following section, we will explain the relevance of this dimension as a socio-emotional skill, as well as methods suited to proxy it without recourse to self-reporting or assessment through past behaviors^[[4]](#footnote-4)^.

**Emotion-detection theory.** The complex task of measuring emotions can be executed using self-reported measures. These methods generally follow the Positive and Negative Affect Schedule^[[5]](#footnote-5)^ ^56^.

As the PANAS is self-reported, it experiences issues similar to those mentioned above for self-reported psychometric tests. In particular, performance can be influenced by the examinees' emotional and motivational states ^22,23,26^, the test setting ^19^, and reference bias ^20,21^. Therefore, an alternative can be the use of neurophysiological methods drawn from affective neuroscience^[[6]](#footnote-6)^.

Over the past decade, emotion-detection research has employed a variety of physiological measurements and methods, including pupil dilation, heart rate, and skin conductance for arousal, and voice and facial manifestations for valence ^17,57-63^. However, many of these measurements can be consciously modified; thus, the signals they produce are not purely objective ^58^. In particular, those related to valence -- e.g., facial expressions -- are easily modifiable, and also unambiguously related to self-reporting, and are therefore the main feature of interest in this study^[[7]](#footnote-7)^. Thus, the use of electroencephalograms (EEGs) becomes particularly appropriate.

The electrodes in an EEG headset capture the electrical activity corresponding to field potentials resulting from the combined firing of many individual neurons in the brain^[[8]](#footnote-8)^. However, cortical activity measures are distorted by the tissue and skull wall between the electrodes and the neurons. This introduces noise and reduces the intensity of the recorded signals. Regardless, EEG measurements offer important insights into the electrical activity of the cortex ^59,60^. Indeed, EEG recordings allow us to measure brain activity and predict emotional states and physiological responsiveness, improving both accuracy and objectiveness in respect to the aforementioned physiological measurements ^17,58-63^.

It is important to note that these EEG recordings are not intended to describe and/or predict personality traits or character. For instance, in a recent study, Korjus et al. ^64^ showed that there is no correlation between resting-state EEG waves and any of the five personality dimensions of the self-reported Big Five Inventory (BFI, ^65^), and concluded that the extraction of personality traits from resting-state EEG power spectra are extremely noisy, if not impossible. In summary, EEGs can relate self-reported tests for non-cognitive skills -- e.g., BFI or Grit scale -- to transient emotional states during testing, but do not enable us to predict psychometric test scores from a normal, resting-state EEG recording without stimuli.

A number of authors have considered diverse methods aimed at recognizing emotions from EEG recordings, improving both the accuracy and objectiveness relative to the aforementioned physiological measurements ^16,17,57,66,67^. Accuracy accounts for true and consistent results (i.e., for the likelihood that the model can correctly predict the elicited emotion, which is known by the characteristic of the stimuli or a self-reported emotional state after a given experiment).

Numerous studies have attempted to compare those diverse methods of emotion recognition, arguing that the most suitable methodology and strategy -- i.e., the one with the highest predictive accuracy -- is the use of EEG recordings ^16,17,57,60,66,67^^[[9]](#footnote-9)^ . For instance, Brown et al. ^59^ estimate an 82% accuracy for arousal and valence; Yoon and Chung ^63^ found a 70% accuracy for arousal and valence; Verma and Tiwary ^60^ found 85% accuracy for arousal, valence and dominance^[[10]](#footnote-10)^.

Furthermore, the “proof-of-concept” experiment that we conducted on graduate students at Columbia University exhibited 79% accuracy for valence ^26^. The recent development of low-cost portable EEG devices offers an unprecedented opportunity to incorporate methodologies from affective neuroscience in social programs evaluation and experiments in the field^[[11]](#footnote-11)^. In a recent study, Martinez-Leon et al. ^68^ compare the quality of data captured by a professional Biosemi Active II^[[12]](#footnote-12)^ and a low-cost Emotiv EPOC^[[13]](#footnote-13)^ headset. The latter is identical to the device used in this paper. Their results are based on the comparison of the success rate of a Brain-Computer Interface (BCI) system. Higher precision and less variance are found on low cost Emotiv EPOC headset datasets. Moreover, the authors conclude that the Emotiv EPOC low-cost headset can be used on motor imagery BCI systems. In summary, a suitable strategy to proxy emotional regulation -- i.e., emotional state and responsiveness -- for dimensions of arousal and positive (approach) and negative (withdrawal) valence (motivation) in lab-in-the-field experiments can be the use of low-cost EEG recordings ^69^.

Matlab 2014b software (Waltham, MA, USA), and EEGLAB open-source toolbox ^70^, were used to conduct the neurophysiological offline data analysis. The EEG signal was passed through a low-pass filter with a 40-Hz cutoff frequency in order to remove noise coming from the power line and artifacts. The frequency of EEG measurements ranged from 1 to 80Hz, with amplitudes of 10 to 100 microvolts ^17^.

A multi-taper Fourier transform for continuous data sets was used. By using a set of tapers, rather than a unique data taper or spectral window, the algorithm reduced the variance of spectral estimate. Moreover, it was particularly effective for short data segments ^71,72^^[[14]](#footnote-14)^. Many authors suggest that a maximum signal duration of approximately 10 seconds would be required if the dependent measure was an EEG, since a longer one could include factors that are distinct from the elicited emotion ^73^. Finally, in order to smooth the exponential nature of EEG signals, we applied a logarithmic power transformation to the data, which is the standard in this literature ^17,60,74^^[[15]](#footnote-15)^.

We decided that the most suitable method to measure emotions from EEG signals was the one implemented by Ramirez and Vamvakousis ^17^. Evidence has shown that the prefrontal cortex -- in addition to the amygdala and the insula -- coordinates consciousness and regulates emotions ^28,75^. Measuring emotions therefore requires consideration of the EEG signals recorded through electrodes AF3, AF4, F3, and F4 in the 10-20 standard classification (see Fig. S1 for the diagram with all 10-20 system locations), which are located on the prefrontal lobe. The frequencies of interest in measuring emotions are both alpha (8 to 12Hz) and beta (12 to 30Hz) waves ^60,73,76-81^^[[16]](#footnote-16)^. Alpha waves are predominant in relaxed states and brain inactivation, while beta frequencies are associated with alertness and an excited state of mind ^17,59-61,63,67,69^.

Consequently, the beta-alpha ratio is an effective indicator of the level of arousal. Therefore, the arousal index can be estimated by the following equation:

$$\begin{matrix} {arousal}_{i}=\frac{\beta_{F,i}}{\alpha_{F,i}} & [1] \end{matrix}$$

where $F$ indicates the simple average of electrodes located on the frontal brain areas AF3, F3, AF4 and F4 in the 10-20 universal system, while $i$ indicates an individual in the sample.

On the other hand, previous neurophysiological evidence has shown that emotional valence has different representation in the brain’s left and right cortical hemispheres ^69,79^[[17]](#footnote-17)^^. A decrease in activity over the frontal right region correlates with positive emotion, while one over the left frontal region is related to negative emotion. As activity decrease -- i.e., inactivation -- could be measured as the inverse of arousal level, it is possible to estimate valence level by the following relation:

$$\begin{matrix} {valence}_{i}=\frac{\alpha_{F4,i}}{\beta_{F4,i}}-\frac{\alpha_{F3,i}}{\beta_{F3,i}} & [2] \end{matrix}$$

where $F3$ and $F4$ indicate electrodes located on the left and right frontal brain areas, respectively. Individuals are indexed by $i$.

As mentioned before, the literature “positive-valence” and “negative-valence” indices as approach and withdrawal motivations to stimuli, respectively ^73,79^. Evidence from the psychology and neurophysiology literature points out that frontal EEG asymmetry is associated with different emotional and psychological states, rather than valence. In a seminal work, Davidson et al. ^82^ suggested a model, called approach/withdrawal theory, to investigate frontal EEG asymmetry during emotional states. He claimed that the left pre-frontal cortex (PFC) activity is involved in a system facilitating approach behavior to positive stimuli, while the right PFC activity participates in a system facilitating withdrawal behavior from aversive stimuli. This model claims that processing related to emotional valence itself is not lateralized in the PFC. Rather, emotion-related lateralization is observed because emotions contain approach and/or withdrawal components.

That is to say, frontal EEG asymmetry is perceived because emotions contain approach and/or withdrawal components. Thus, emotions will coexist with either right or left asymmetry, whether they are accompanied by approach or withdrawal behavior ^15,73,79,82^. Approach/withdrawal motivational states have frequently been linked to asymmetries in left/right frontal cortical activation, especially using EEGs, although meta-analyses of Functional magnetic resonance imaging (fMRI) data have failed to find consistent localizations ^83^.

Finally, there are a number of papers that consider the left, relative to right, frontal cortical activity (LFA) to build behavioral indices of approach or motivation. For instance, Hughes et al. ^9^ examined the relation between LFA and effort expenditure for reward, a behavioral index of approach motivation. They found that subjects with greater resting LFA were more willing to expend greater effort in the pursuit of larger rewards, particularly when reward delivery was less likely.

**Electroencephalogram (EEG) measurement.** The EEG recordings were often conducted in the Neurophysiological experiment. EEG devices measure the voltage change that occurs when a neuron fires. When a positive change in the voltage crosses a certain threshold, an action potential is triggered, causing the voltage to increase from the resting potential of about -60mV to +20mV. This electrical activity generated by a group of neurons is measured over the cortex with EEG electrodes.

While participants took the aforementioned tests in each experiment, their brain activity was measured using the low-cost, portable Emotiv EPOC EEG headset. The EEG recordings were then transmitted wirelessly to a laptop computer. The online data were processed using OpenViBE ^84^. Moreover, MATLAB 2014b, 2020b software (Waltham, MA, USA), and the EEGLAB open-source toolbox ^70^ were used to perform offline data analysis.

Using Fourier frequency analysis, the original signal was converted to the frequency domain to select data in the frequency bands of interest and was then transformed back to the time domain. Many authors suggest that a maximum signal duration of 10 seconds is necessary for EEG measurements in order to exclude factors distinct to the elicited emotion ^73^. Here, we consider an epoch window of 5 seconds for the analysis.

In conventional spectral analysis, the spectrum of a time-series $x(t)$ observed in a time-window of duration $T$ is estimated as the square of the absolute value of its tapered Fourier transform. Namely:

$$\begin{matrix} S_{conv}\left( f \right)=\left| X(f) \right|^{2}=\left| \int_{0}^{T} h(t)x(t)e^{2\pi ift}d\tau\right|^{2} & [3] \end{matrix}$$

where $h(t)$ is a taper or windowing function. Some standard choices for the windowing function are the constant, the Hanning window, the Hamming window, and the Parzen window ^85^.

The standard method has three main caveats ^85^. First, the choice of the type of windowing function is arbitrary. Second, the estimate is biased -- i.e., the expectation of the spectral estimate differs from the population spectrum -- for any type of windowing function. Third, the spectral estimate has a higher variance than that found using the multi-taper method^^[[18]](#footnote-18)^^. Thus, in order to address the limitations of the standard method, the multi-taper Fourier transform is used for continuous data sets.

Instead of choosing an arbitrary windowing function, the multi-taper method finds W for windowing functions that are maximally concentrated within $\left[ -W,W \right]$, which are known as Slepian sequences ^85^. For duration $T$, around $K=2TW-1$ of these functions can be found ^85^. Therefore, for $W>1/T$, there are multiple such functions that are well-concentrated in frequency and therefore have bias-reducing characteristics. The multi-taper estimate of the spectrum is given by using each of these tapers as windowing function and averaging the resulting spectral estimates, as the following equation illustrates:

$$\begin{matrix} S_{MT}\left( f \right)=\frac{1}{K}\sum_{k=1}^{K} \left| X_{k}(f) \right|^{2}=\frac{1}{K}\sum_{k=1}^{K} \left| \int_{0}^{T} u_{k}(t)x(t)e^{-2\pi ift}d\tau\right|^{2} & [4] \end{matrix}$$

where $u_{k}$ are the Slepian sequences or tapers. By using a set of tapers, rather than a unique data taper or spectral window, the algorithm reduces the variance of spectral estimate, due the orthogonality of the Slepian sequences. Moreover, it is particularly effective for short data segments ^71^[[19]](#footnote-19)^^.

Finally, in order to smooth the exponential nature of EEG signals we apply a logarithmic power transformation to the data, which is the standard in this literature ^17,60,74^. EEG activity is typically studied in the 1 to 80Hz range, with amplitudes of 10 to 100 microvolts ^17^. Here, the EEG signal was passed through a low-pass filter with a 40Hz cutoff frequency in order to remove noise from artifacts. The alpha (8 to 12Hz) and beta (12 to 30Hz) bands were described as particular areas of interest for emotion recognition for both valence and arousal. The influence of eye movement or blinking artifacts is most dominant below 4Hz, while heart related movements, i.e. Electrocardiogram (ECG), artifacts around 1.2Hz, and facial muscle movements, i.e. electromyogram (EMG), artifacts above 30Hz ^61^. Therefore, by extracting only the alpha and beta frequencies, the influence of much noise is already significantly reduced. Lastly, arousal and valence indices were constructed following the equations [1] and [2], respectively.

**From neuro-physiological indices to measuring emotional regulation.** EEG readings are not meaningful without some means of interpreting the data they produce. We applied emotional detection theory, taken from the affective neuroscience literature, to widely used neurophysiological markers, allowing us to interpret EEG readings as measures of participants’ emotional states and responses ^9-18^.

We have explained that self-reported measures are suboptimal for estimating the impact of interventions because they can be affected by other factors that may impact individuals' emotional states and be confounding with the treatment itself. As such, emotional regulation can be proxied using emotion-detection theory, allowing us to interpret EEG recordings to determine the presence of emotional regulation. In this study, we use the James-Lange theory of valence and arousal, which is widely used as a simple and schematic model of emotions in computer neuroscience ^17,60,73,79^. In this model, arousal is a proxy of an individual's stress, estimated directly from subject’s brain activity. Valence can be interpreted as a positive or negative mood, as well as an attitude of either approach toward or withdrawal from a stimulus ^73,79^.

In addition, other scholars have used this same measure of valence -- that is, left relative to right frontal cortical activity (LFA) -- to build emotion-related (or approach/motivation-related) indexes and correlate them with behavioral or performance outcomes ^9-14,17,18,86^. For instance, Hughes et al. ^9^ examined the relation between LFA and effort expenditure for reward, a behavioral index of approach motivation. They found that subjects with greater resting LFA were more willing to expend greater effort in the pursuit of larger rewards, particularly when reward delivery was less likely.

To generate these measures, we follow the methodology developed by Egana-delSol ^26^. We acquired portable Emotiv EPOC EEG headsets to obtain a proxy measure of students' emotional states and responsiveness to stimuli in the arousal-valence locus ^87^. These devices provide an average accuracy or correct detection that is similar to research-grade instruments and an emotion-detection accuracy of about 79% within subjects ^26,68,86,88-92^[[20]](#footnote-20)^^.

Then, we established a lab-in-the-field setting to collect three streams of neurophysiological data for each student: 1) pretest resting emotional state from EEG recordings; 2) psychometric tests to measure noncognitive, creative, and cognitive skills; 3) emotional responsiveness to both positive and negative stimuli (see Fig. S2 as a reference of the lab-in-the-field setting).

In the first stage, the pretest resting emotional state, we collected EEG recordings to estimate emotional arousal and valence indexes at resting state while students watched a black cross in the center of a gray screen for a period of 30 seconds. We are aware that the conditions in the field are sub-optimal when comparing to the lab in terms of having a constant lighting, the same “gray” because of the latter, etc. In our favor, we can argue that we are collecting data from around 600 participants, comprising many different situations; thus, on average, the nuances should cancel out between both subjects within a group and the treatment and control groups.

In the second section of data collection, students responded to a battery of psychometric tests, which included the Rotter Locus of Control Scale ^93^, Raven-like progressive matrices, and the Cognitive Reflection Test (CRT). Our measure of locus of control indicates children think they are unable to control what happens in their lives. More specifically, a decrease in locus of control indicates that students feel they can manage their experiences, thus demonstrating an increase in self-efficacy. Raven is a measure of abstract reasoning and a nonverbal estimate of intelligence. It is implemented as a set of matrices in progressive order. Finally, CRT is a test designed to measure if an individual tends to automatically choose an initially incorrect response and then engage in deeper reasoning to find a correct answer.

During the third stage, right after the students finished the battery of psychometric tests, we obtained emotional response intensity for negative and positive stimuli in terms of valence locus. Here, we exposed students to alternate series of images selected to elicit positive and negative emotional responses in order to estimate post-stimuli valence indexes. The images were taken from the Geneva affective picture database (GAPED)^^[[21]](#footnote-21)^^. The data here is epoch considering 5 seconds since the onset of the stimuli.

Then, using this response intensity measure and the valence-at-resting-state index from the first stage, we estimated emotional responsiveness as the difference between both levels. Specifically, the positive (negative) valence difference indices measure the variation in valence index recorded in the third stage when the stimulus was positive (negative) net of the individual's baseline resting state valence index recorded in the first stage. Both differences can be interpreted as a lower level of overreaction of participants -- they become more phlegmatic.

**Attrition: additional tables.** Here, we present the four additional models discussed above that contribute to rule out different sources of bias due attrition.

First, Table S1 presents a regression of the attrition dummy -- i.e., a variable that is equal to 1 if students participate only in the baseline study and 0 otherwise -- on the treatment dummy; it shows no difference in the likelihood of attrition between the treatment and control groups. The same is true when the dummy is defined as equal to 1 if students participate only in the follow up and 0 otherwise. Additionally, Table S2 shows that bounding the treatment effects on the variables using Lee bounds ^94^ reveals that zero belongs to the treatment effects interval for all variables on both only at baseline and new at follow-up comparison groups. Thus, attrition is unlikely to have biased the results reported below.

Thus, all in all, this evidence suggests that attrition is unlikely to have biased the results reported.

**Minimum detectable effects.** In order to accurately distinguish between true null effects and results that fail to reach significance but for which we cannot confidently rule out treatment effects, we calculated the “Minimum Detectable Effect (MDE) Size” for our experiment.

The MDE added strength to our study by providing an objective metric by which we were able to assess the minimum improvement over the baseline to which we were willing to assign statistical significance. In the case of this study, we reported an MDE with an 80\% power at a significance level of 0.05. This is illustrated by the equation below.

$$\begin{matrix} MDE=(t_{1-k}+t_{\frac{\alpha}{2}})\times\frac{\sigma}{\sqrt{NP(1-P)}} & [5] \end{matrix}$$

where $t_{1-k}=0.84$ and $t_{\frac{\alpha}{2}}=1.96$ are the t-statistics required to obtain 80% of power and 0.05 significance level ($\alpha$), respectively. The expression $\frac{\sigma}{\sqrt{NP(1-P)}}$ identifies the standard error of the treatment coefficient considering the fraction $P$ of the sample population that was treated.

Following Haushofer and Shapiro ^95^, we used this formula to computes the MDE for null results. MDEs were reported for each model.

**Multiple hypothesis testing.** In addition to correct the p-values of our mechanisms using Wild bootstrap because of the relatively small number of clusters in our study, we correct our p-values to account for multiple hypothesis testing. In our study we have only two main educational outcomes -- i.e., high school dropouts and registration to PSU -- that were selected given the availability of administrative data. However, we considered many different channels to work as mechanisms of the impact observed. Regardless some authors argue that it is not necessarily useful to correct p-values when trying to elucidate mechanisms, we conducted a multiple hypothesis testing correction on our potential mechanisms organized by three main groups/families: Socio-emotional skills and Emotional Regulation. For cognitive skills we do not correct p-values because we have only one measure. In particular, we use the free step-down resampling method for multiple hypotheses testing to adjust the family-wise error rate (FWER). Specifically, the Stata command ‘wyoung’ is used developed by Jones et al. ^96^. We conducted 10,000 simulations for each multiple hypotheses testing with same covariates and specification of the results presented in the results section. In order to make our multiple hypotheses testing results replicable, we use the seed number of 20 ^^[[22]](#footnote-22)^^. As Table S3 shows, only valence at baseline became non-significant after correcting by multiple hypothesis testing under the Sidak-Holm method.

**Educational outcomes and EEG features.** The Probit model of EEG features on PSU registration variance and ratio is test by PSU registration status. The Probit model ^97^ is defined by the following equation:

$$\begin{matrix} y^{*}=X\beta+\varepsilon& [6] \end{matrix}$$

$$\begin{matrix} y=\left\{ \begin{aligned} \begin{matrix} 1 & y^{*}>0 \end{matrix} \\ \begin{matrix} 0 & otherwise \end{matrix} \end{aligned} \right.,\varepsilon\sim\eta(0,1) & [7] \end{matrix}$$

where $y$ is the variable corresponding to registration for the PSU ($y=1$ if the student had registered to take the high-stake examination) and $X$ is the matrix of individual emotional biomarkers from EEG recordings^^[[23]](#footnote-23)^^.

As shown in Table S4, EEG features did not correspond to a linear relationship to our outcomes of interest. This result may be due to the more general non-linearity in the relationship between EEG features and educational outcomes. In fact, the Models (2) and (3) in Table S4 show significant marginal effects for the quadratic expression of EEG features; in particular, valence at baseline (no stimuli) and when facing negative stimuli. Moreover, both the Akaike and Bayesian Information Criteria support the argument that the EEG features added information to the model, as Model (3), which includes only significant variables in order to minimize the penalization for over-fitting, informs^^[[24]](#footnote-24)^^. These results are consistent with the non-linear relation between EEG features and outcomes that we have been suggesting exists. Additionally, the results of ratio test by PSU registration status are shown from Table S5 to S10.

**Fragments not to be considered.** We summarize our findings here and then present results and discuss their broader implication. First, we find significant effects of the program on educational outcomes, socio-emotional skills, and emotion regulation. We find that the program decreased high school dropout rates by approximately six percentage points, which is economically relevant. The intervention also increased the probability of registering for the Chilean college entrance exam (PSU) by 13 to 38 percentage points^^[[25]](#footnote-25)^^. Although registration for the PSU is voluntary, it is a requirement to enroll in any college. Therefore, one could interpret our finding as showing an effect on expectations or aspirations about the future^^[[26]](#footnote-26)^^.

We do not find a significant impact of the program on self-reported socio-emotional skills measures, which is consistent with previous related studies ^20,40,41,98,99^. These null results were expected and documented before running the RCT^^[[27]](#footnote-27)^^.

In order to collect the data for our study, we built a portable laboratory containing seven workstations, each of which consisted of a low-cost portable EEG headset paired with a high-capacity laptop. We discuss the headset used in greater detail in a later section, as well as the theory involved. It is again worth stressing that while the use of EEG recordings may seem speculative to many in the field, we endeavored to adhere to well-established norms and best practices in the fields of neurophysiology and computer neuroscience. To collect neuro-physiological measures potentially associated with emotional regulation and stress, we focused on changes in the alpha and beta frequencies of the brain’s prefrontal cortex, observed in a resting state (with no stimuli) and under emotionally laden stimuli. Following the James-Lange theory of valence and arousal, we recorded the brain activity of a random subsample of enrolled students before and after the participation in the program in a lab-in-the-field setting^^[[28]](#footnote-28)^^. The EEG devices and the experimental paradigm were piloted before going to the field in a lab setting at the Laboratory for Intelligent Imaging and Neural Computing (LIINC) at Columbia University ^26^.

Materials and Methods

**Geographic location and the portable laboratory in the field.** Fig. S3 shows the geographical distribution of both treated and control group school. We built a portable laboratory containing seven workstations, each of which consisted of a portable EEG headset paired with a high-capacity laptop. This experimental setting, which had previously been piloted at Columbia University, enabled us to obtain a proxy measure of our subjects' emotional regulation capabilities in a non-lab context as is depicted in Fig. S2. The circumstances of data collection were homogeneous between treatment and control schools. Thus, it is reasonable to assume that there is no differential noise contamination to the data between treatment and control groups (e.g., external noise, time of the day, breakfast content, room lighting). We set up our portable labs in empty classrooms using white curtains to prevent direct external light in order to keep the contrast level similar across schools and subjects.

**Timing.** We illustrate the timeline of the experiment in Fig. 1 in the main text. The intervention began in March 2015, when we conducted the baseline data collection. We brought the portable neuro-scientific lab to all eight schools during this time. The follow-up data collection happened when the second semester started, in August of the same year. Thus, treatment effects correspond to participation in the entrepreneurship program during one semester.

**Data Collection.** We use EEG recordings, to construct an objective instrument that measures emotional regulation skills. Specifically, we employ the James-Lange theory of valence and arousal, which relies on neurophysiological measurements, to accurately capture and codify emotional data. This theory (2D VA space) is depicted in Fig. S4^[[29]](#footnote-29)^ ^26^. This model argues that there is a correlation between physiological experience (i.e., feelings) and emotions. It is important to note that these EEG recordings are not intended to describe and/or predict personality traits or character. For instance, in a recent study, Korjus et al. ^64^ show that there is no correlation between resting-state EEG waves and any of the five personality dimensions of the self-reported Big Five Inventory (BFI, ^65^), and concluded that the extraction of personality traits from resting-state EEG power spectra are extremely noisy. In summary, EEGs can relate self-reported tests for non-cognitive skills (e.g., BFI or Grit scale) to transient emotional states during testing, but do not enable us to predict psychometric test scores from a normal, resting-state EEG recording without stimuli. As illustrated in *Fig. 1* of main text, our experiment was primarily concerned with the collection of three sets of data: 1) pre-test resting emotional state from EEG recordings; 2) a battery of psychometric tests; and 3) emotional responsiveness to both positive and negative stimuli.

The experiment diagram is shown in Fig. S5. The pre-test resting state is measured with EEG scalp recording when the students watched a black cross in the center of a gray screen. After the pre-test resting state emotional indices measurement, we applied a psychometric test that includes the Grit scale, the locus of control scale, the raven-like progressive matrices, and Torrance's test of creative thinking. We measured the participants' emotional responsiveness immediately after the students had finished this test regimen. The experiment consisted of showing an alternating series of 5 positive and negative images in order to elicit emotional responses from each participant, which we illustrate in Fig. S5. These reactions were then converted to valence indices as methods introduced in Neurophysiological Measurements and Experiments section of main text (see more technical details in **Emotion-detection theory** section of **Supplementary Information Text**). There are a number of papers that consider valence, or LFA cortical activity, to build behavioral indices of emotions or motivation. For instance, Hughes et al. examined the relationship between valence and effort expenditure for reward, a behavioral index of approach motivation ^9^. They find that subjects with greater resting state valence were willing to expend greater effort in the pursuit of tasks with larger rewards, particularly when reward delivery was less likely. We discuss this literature in more detail in **Emotion-detection theory** section of **Supplementary Information Text**.

**Quality of the Experiment.** (1) Baseline Balance: This subsection presents the descriptive statistics and attrition rates of our field experiment. We show in Table S11 that we cannot reject similarity between treatment and control groups for all variables at baseline (i.e., March 2015). For the difference in means test we report in Table S11 we cluster standard errors at the school level to control for unobserved shocks that may be correlated within schools. We still include baseline measures as controls in our main specification to increase statistical power.

(2) Compliance: Due to the nature of this RCT, we did not encounter any problems of compliance; everyone in our treatment group was treated, whereas no one in the control group was. This is because the treatment was at the school level, considered all students who were attending 12th grade of secondary, and the program was conducted during regular school hours where attendance is mandatory by law until the age of 18. The only scenario in which a student could leave the treatment group would be by moving to a different school or dropping out altogether.

(3) Attrition: In this section we discuss the sources of attrition in our experimental context, and whether there are differential rates of missing data between treatment and control groups. We identify five sources of data attrition in this study. We discuss below whether they compromise the causal interpretation of our estimates.

First, experimental attrition due to inaccurate identifiers prevented some students from being linked to the administrative data. This attrition is due to privacy restrictions from the Institutional Review Board at Columbia University. Field experiments were implemented using several working stations -- i.e., a laptop with an EEG headset. Six and eight stations were used in both the baseline and follow up, respectively. When conducting the experiment, several difficulties were encountered in linking individual's metadata to their respective computer and in matching written records to the data files of individuals using date of file creation, modification, and other metadata related to files. As Table S12 shows, this problem accounts for around 5% of the total attrition (from 195 to 180 and 136 to 116 in the baseline and follow up experiments, respectively).

Second, we find some degree of attrition due to non-participation in the follow-up round of measurements. This resulted in a decrease of approximately 28% in the sample size (from 287 to 205). We do not find a statistically significant difference in baseline characteristics between treated individuals that participated only in the baseline testing and those who participated in both baseline and follow up (see Table S1).

A third source of attrition arises because of the quality of data recordings. EEGLAB (i.e., the Matlab toolbox used to analyze EEG data) sometimes failed to read the EEG recordings properly. We faced two problems: very long or dense hair that avoids the proper operation of the EEG headsets, and freezing computers^[[30]](#footnote-30)^. Moreover, we drop some observations after signal processing (i.e., high and low band-pass filtering), which is common practice in the literature. This leads to a relevant decrease in the final sample (i.e., from 287 to 195 and from 205 to 136 in the baseline and follow up, respectively). Nonetheless, this source of attrition is not particularly concerning because it happened ex-post participation in the surveys.

Fourth, it was also not possible to rule out the attrition due to any potential un-observable characteristics that could be present in a major proportion of the group of students who participated in the follow up. Anecdotally, evidence obtained from teachers was also helpful in interpreting the causes of much of the attrition that occurred between baseline and follow-up testing. For example, many students lacked motivation and, in particular, did not want to attend school in the first place. Further, many of the control schools' students who had participated in the baseline experiment were absent from school on the day of the follow-up. In extreme cases, some students dropped out of school altogether between the two rounds of testing. This could be due to, for example, the degree of intrinsic motivation. The locus of control test would capture this possibility, and the results would be different between the two groups following treatment even though these differences are not observable at baseline (see Table S11). However, we do not observe significant differences between groups on locus of control in the follow up, providing evidence against the idea that students in the treatment group were overall less engaged in the experiment.

Fifth, boredom may likely have generated attrition by contributing to the lack of incentive to participate in the study, especially in the control group (whose participants had no formal relation to the program). Students in the control group had not been made privy to any high-level details of the experiment, and the baseline study could have easily been boring for young students; the experiment lasted for 30 to 40 minutes on average, including setup, and actual testing required a further 20 minutes. The relatively large number of participants -- before considering whether they had a valid EEG data file, see Table S12 -- in the control group at baseline (164 subjects) was unexpectedly high compared with that of the treatment group (123 subjects).

In Table S13, we compare observable characteristics between treatment and control groups individuals who participated in the baseline round but did not participate in the follow-up. In Table S14, we compare treatment and control students who were new at the follow-up. From the statistics reported in these tables, we conclude that differential attrition does not seem to invalidate the causal interpretation of the outcomes that result from this experimental design.

A regression of the attrition dummy considering those students who had participated only in the baseline conducted on the treatment dummy showed no difference in the likelihood of attrition between the treatment and control groups. The same proved to be true when we considered those who had only participated in the surveys and neuro-physiological experiments in the follow up (see **Attrition: Additional Tables** section in the **Supplementary Information Text**). Unfortunately, we did not have information at baseline for the “only at follow up” comparison group, thus, we were not able to test their differences in observable variables before the program started. Regardless, we should still expect differences in outcomes at follow up between treatment and control groups.

Finally, bounding the treatment effects on the variables using Lee bounds ^94^ reveals that zero belongs to the treatment effects interval for all variables on both only at baseline and new at follow-up comparison groups. Thus, attrition is unlikely to have biased the results reported below (see **Attrition: Additional Tables** section in the **Supplementary Information Text**).

**Robustness Checks: Educational Outcomes and EEG Indices.** This section provides empirical evidence showing that educational outcomes correlate with EEG biomarkers -- i.e., valence and arousal indices. This provides support for the claim that emotional regulation, which is proxied through EEG recordings, is the channel through which the program affects educational outcomes.

Our main analysis focuses on registration for the PSU as the main outcome. We provide additional evidence that EEG features and registration for the PSU are empirically related in a non-linear manner, as depicted by Fig. S6. There is no statistically significant linear correlation between EEG features and registration for the PSU. Pair-wise correlation and ANOVA tests failed to reject the null hypothesis of no significant correlation. The presence of outliers, which is insinuated from the box plot presented in Fig. S7, is a crucial caveat on ANOVA testing ^97^.

Second, we analyze the distribution of different EEG features conditional on the individual's registration status for the university entrance exam, the PSU. We show in Fig. S6 that these conditional distributions are different, especially with respect to second and higher moments. In fact, we run a variance ratio test between different EEG indices of emotions, and we show in Tables S9 and S12 that we can reject the null hypothesis that these variances are equal. These results support the claim that the program impacts outcomes, and that one of the mechanisms by which it does so is by affecting the EEG features of emotional regulation skills. We also estimate the probability to register for the university entrance exam based on EEG features using a Probit model. We conclude that the EEG measurements are relevant to predict registration for the PSU.

Finally, based on the EEG recordings, we estimate the impact of the entrepreneurship program on the variance of emotional biomarkers. The program impacted the EEG features of emotions. In particular, it impacts valence and arousal at baseline, and the reaction to negative stimulus conditional on baseline measures. Since we find evidence that the variance of EEG measurements is correlated with educational outcomes, we also test for such non-linearity in the effect of the entrepreneurship program on emotional regulation. We reject the hypothesis of equal variance between treatment and control groups for the response to positive and negative indices, which we report in Tables S5 to S8. This reinforces the evidence that emotional regulation and educational outcomes are related.

In summary, the data suggests that there is an empirical relationship between EEG features based on emotion-detection theories and educational outcomes. For the purpose and scope of this study, the non-linear relationship that has been shown is sufficient to argue that emotional regulation is a possible mechanism for explaining the impact of programs aimed at improving socio-emotional skills. Future work can advance the understanding of the non-linear relationship between the EEG features of emotional regulation and educational and labor market outcomes.

**Accounting for Small Number of Clusters and Multiple Comparisons.** Previous work suggests that clustering at the level of treatment is appropriate in designs such as this. In our setting, we would therefore cluster at the school level. However, given the few clusters considered in our randomization, we estimated the exact p-values that are the result of 1,000 replications of a Wild bootstrap following Webb's algorithm for few clusters ^97^. Exact p-values are reported when possible in each table for the treatment variables.

In general, false positives become a concern when conventional statistical methods are relied upon to interpret the results of studies, such as ours, in which a number of outcome variables are investigated. In many cases, researchers have remedied this issue by way of measures such as “Family Weighted Error Rate (FWER)” ^95,100^, or through the creation of outcome indices ^101,102^ that enable them to arrive at more precise measures of statistical power, significance intervals, and error rates in such settings.

Although our own study examined a number of mechanisms of the observed impacts, we opted to use significance ratings using Wild bootstrap. This was a viable approach for the study at hand for two main reasons. First, our study did not rely on “composite” or index variables to determine outcomes. As mentioned earlier, studies such as ours traditionally rely on univariate proxies to measure the particular mechanism in question. Second, the purpose of studying individual variables within family groups is to understand mechanisms, rather than to isolate individual variables for the purpose of generalizing a given set of conclusions ^95^. Nevertheless, as a robustness check, we conducted a multiple hypothesis testing correction on our potential mechanisms organizing them in two main groups/families: Socio-emotional skills and Emotional Regulation. Only valence at baseline became non-significant after correcting by multiple hypothesis testing under the Sidak-Holm method (see the Table S3 and the **Multiple Hypothesis Testing** section in the **Supplementary Information Text** above for more details).


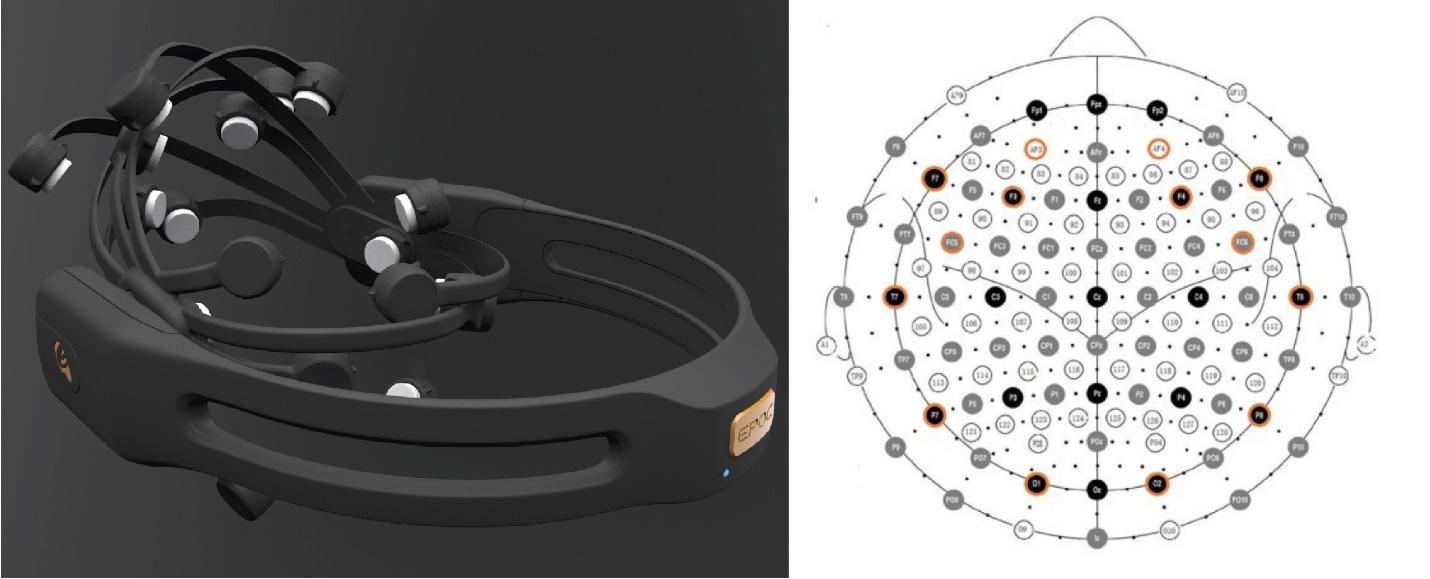


Fig. S1. Emotiv EPOC Device (left) and the 10-20 Location System of Electrodes (right).


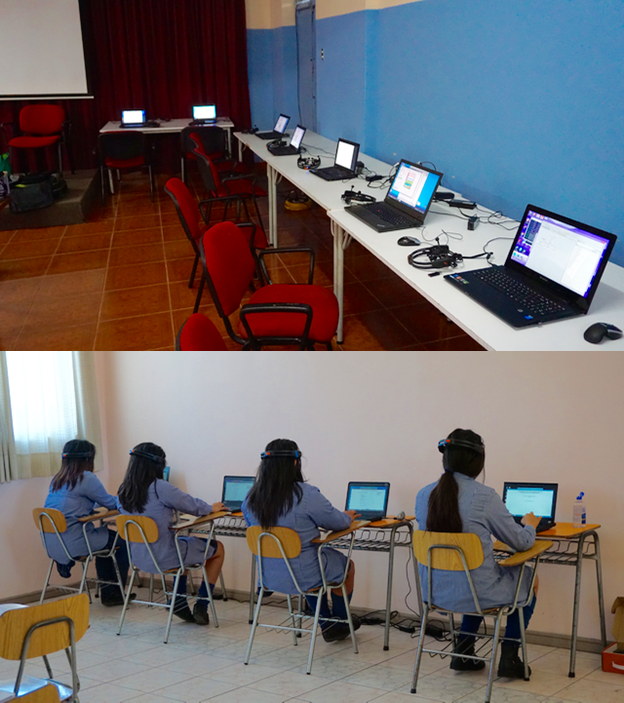


Fig. S2. Field Experiment Setting. Each workstation includes both EEG headset paired with a high-capacity laptop. Each participant will use individual setup during experiment.


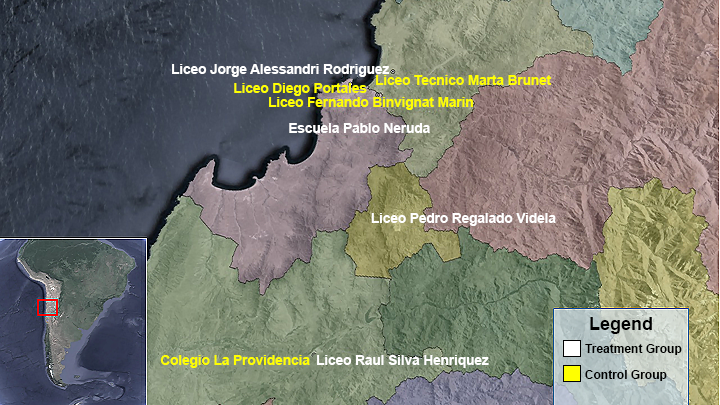


Fig. S3. Geographic Location of Schools in the Field Experiment. Eight different locations are randomly selected, and equivalently separated (4 vs 4) as treatment group (i.e., with entrepreneurship program) and control group (i.e., without entrepreneurship program).


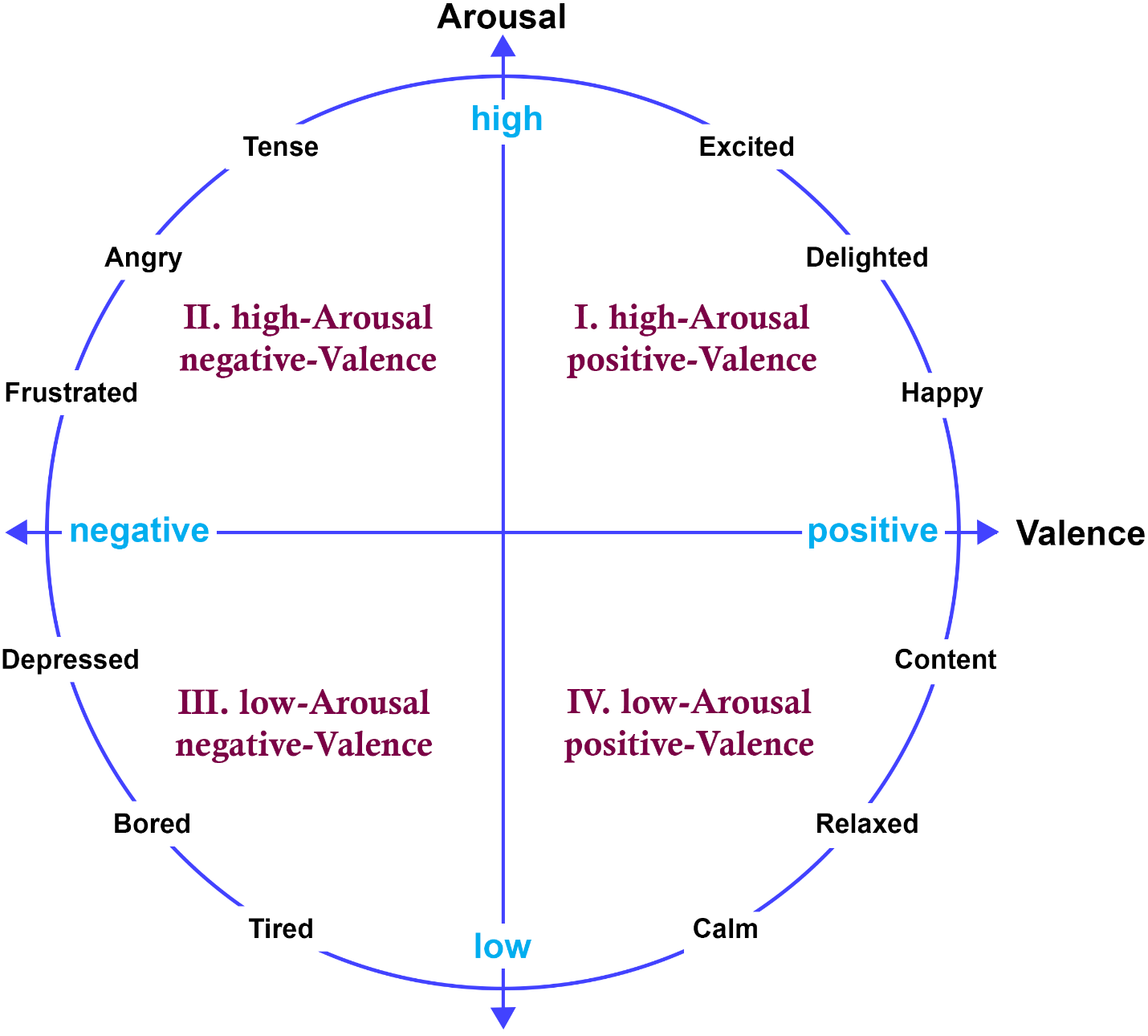


Fig. S4. 2-D Valence-Arousal (VA) space based on James-Lange's theory of valence and arousal ^103,104^. In general, different emotions can be classified into VA space's four quadrants, where first quadrant means high-arousal and positive-valence, second quadrant means high-arousal and negative-valence, third quadrant means low-arousal and negative-valence, and fourth quadrant means low-arousal and positive-valence.

.


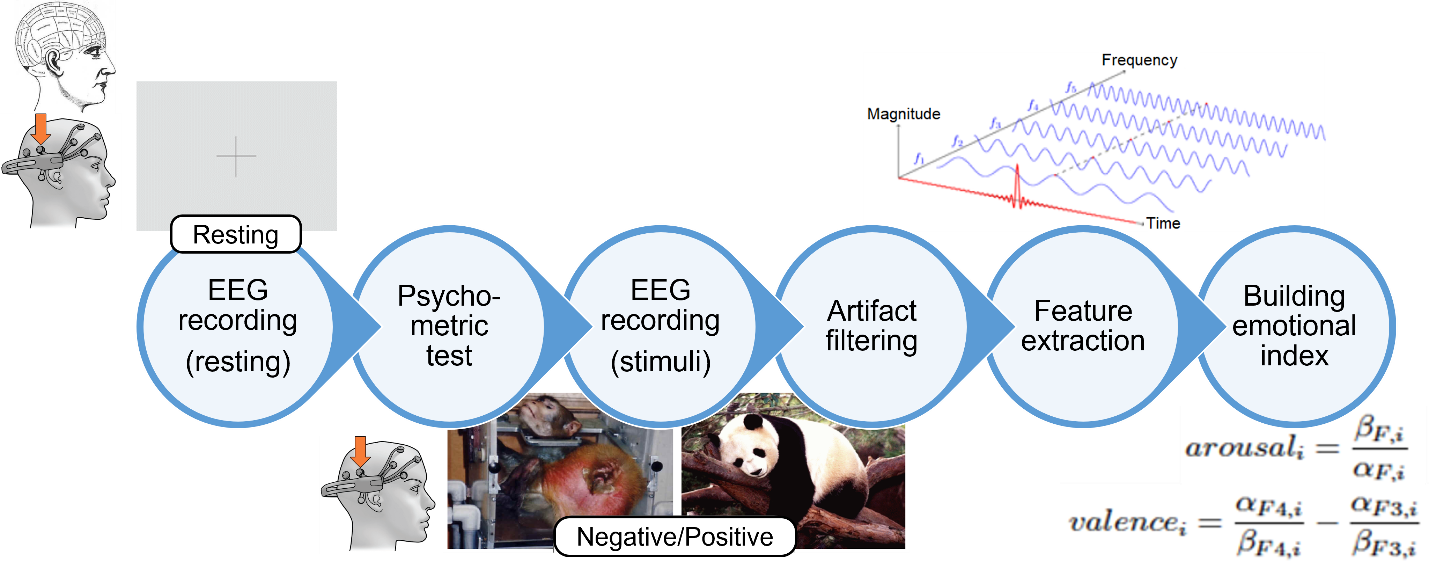


Fig. S5. Experiment Paradigm and Data Processing. Psychometric test is conducted after the EEG recoding with no stimuli (resting state) and followed by the EEG recording with stimuli (5 positive and 5 negative stimulus). Arousal and valence index are calculated based on EEG features.


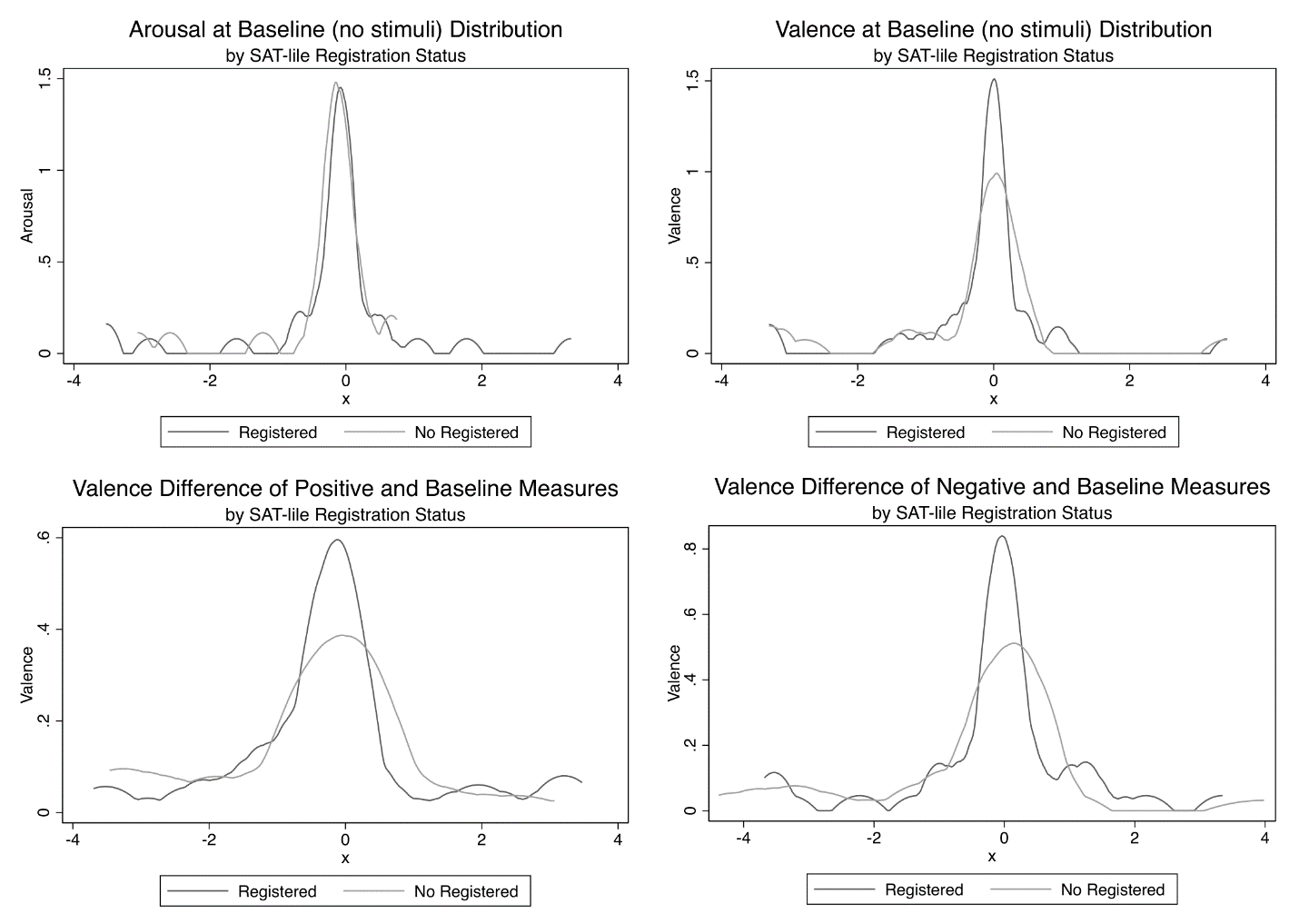


Fig. S6. Distribution of EEG Features by Registration to PSU status. Four conditions are showed here, which are arousal at baseline (no stimuli), valence at baseline (no stimuli), valence difference (positive stimuli) and valence difference (negative stimuli), respectively.


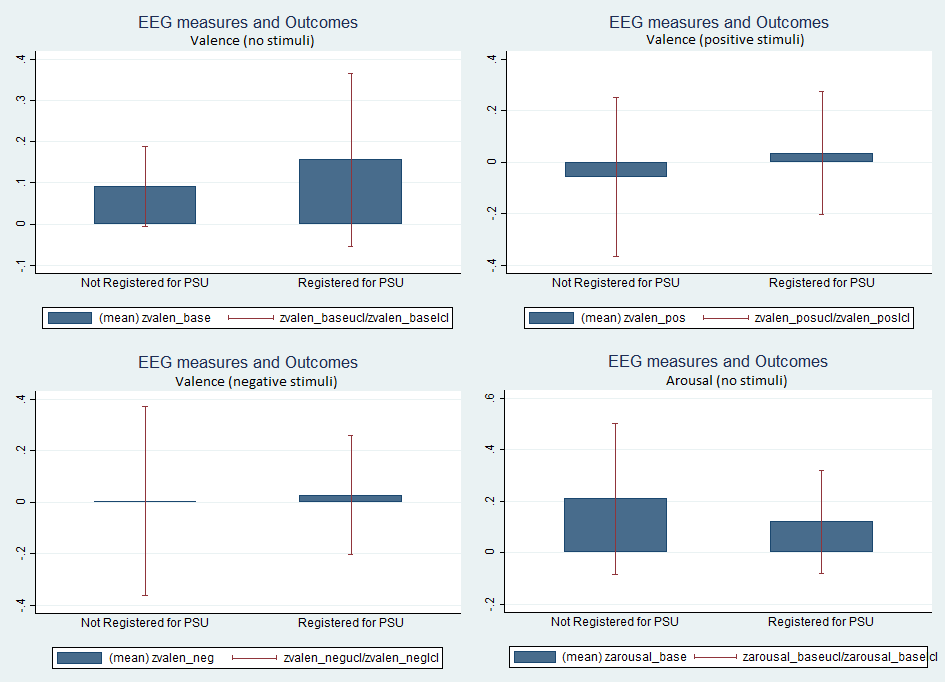


Fig. S7. EEG features and Educational Outcomes. Mean and 95% confidence interval (CI) of four conditions are shown, where mean is indicated by blue box and 95% CI is indicated by red bar.

|  | (1) | (2) |
| --- | --- | --- |
| Variable | Only baseline | Only follow-up |
| Treatment | -0.180 | 0.033 |
|  | (0.116) | (0.197) |
| 12^th^ Grade (=1) | 0.238 | 0.196 |
|  | (0.073) | (0.181) |
| Woman (=1) | 0.022 | -0.002 |
|  | (0.116) | (0.045) |
| Constant | 0.529 | 0.258 |
|  | (0.063) | (0.049) |
| Observations | 180 | 116 |
| R-squared | 0.139 | 0.243 |
| School-level clustering | Yes | Yes |

Table S1. Attrition: treatment effect on baseline only and follow-up only. Robust standard errors in parentheses (*** indicates p<0.01, ** indicates p<0.05, * indicates p<0.1).

| Variables | Lower |  | Upper |  | Observations |
| --- | --- | --- | --- | --- | --- |
| Locus Control Test | 0.098 | (0.207) | 0.565*** | (0.192) | 296 |
| Grit Test | 0.151 | (0.182) | 0.527*** | (0.165) | 296 |
| Creativity | -0.228 | (0.160) | 0.091 | (0.188) | 296 |
| 13 Forces Creativity | -0.155 | (0.178) | 0.185 | (0.173) | 296 |
| Arousal RestState | -0.337*** | (0.146) | 0.016 | (0.118) | 296 |
| Valence RestState | -0.438** | (0.215) | 0.156 | (0.197) | 296 |
| Valence Positive | -0.325* | (0.185) | 0.263 | (0.197) | 296 |
| Valence Negative | -0.525*** | (0.186) | 0.016 | (0.212) | 296 |

Table S2. Attrition: Lee Bounds. Robust standard errors in parentheses (*** indicates p<0.01, ** indicates p<0.05, * indicates p<0.1).

| Mechanisms | Westfall and Young (1993) | Sidak-Holm |
| --- | --- | --- |
|  | p-value | p-value |
| *Socio-emotional Skills* | | |
| Locus Control Test | 0.91 | 0.94 |
| Grit Test | 0.94 | 0.94 |
| Creativity | 0.91 | 0.94 |
| 13 Forces Creativity | 0.91 | 0.94 |
| *Emotional Regulation* | | |
| Arousal RestState | 0.03 | 0.09 |
| Valence RestState | 0.03 | 0.12 |
| Dif Valence Positive | 0.06 | 0.24 |
| Dif Valence Negative | 0.03 | 0.08 |

Table S3. Multiple hypotheses testing p-values correction.

|  | Model (1) | Model (2) | Model (3) |
| --- | --- | --- | --- |
| Variables | Registered for PSU (=1) | Registered for PSU (=1) | Registered for PSU (=1) |
| Arousal Rest | 0.012 | 0.018 | - |
|  | (0.032) | (0.035) | - |
| Valence Rest | 0.029 | 0.020 | - |
|  | (0.058) | (0.065) | - |
| Arousal Rest $\times$ Arousal Rest | - | 0.013 | - |
|  | - | (0.012) | - |
| Valence Rest $\times$ Arousal Rest | - | 0.122*** | 0.090*** |
|  | - | (0.046) | (0.035) |
| ValPosDif $\times$ ValPosDif | - | -0.013 | - |
|  | - | (0.017) | - |
| ValNegDif $\times$ ValNegDif | - | -0.094*** | -0.077*** |
|  | - | (0.032) | (0.028) |
| Observations | 201 | 201 | 201 |
| School FE | No | No | No |
| AIC | 253 | 255 | 246 |
| BIC | 272.9 | 294.7 | 262.5 |

Table S4. Probit Model: Registration to PSU and EEG Features. Robust standard errors in parentheses (*** indicates p<0.01, ** indicates p<0.05, * indicates p<0.1). Note: legal restrictions on privacy issues imposed by the DEMRE-University of Chile in order to allow the merging of the data set of this study with their administrative data made it possible to only identify and control for one school in our sample; legal restrictions on privacy issues imposed by the DEMRE-University of Chile in order to allow the merging of the data set of this study with their administrative data made it possible to only identify the non-municipal school in our sample; variables are defined in the same way as in equation [2] of main text.

| Group | Obs | | Mean | Std. Err | Std. Dev. | | 95% CI  (lower) | 95% CI  (upper) |
| --- | --- | --- | --- | --- | --- | --- | --- | --- |
| Control | 18 | | -0.043 | 0.296 | 1.257 | | -0.668 | 0.583 |
| Treated | 64 | | -0.250 | 0.131 | 1.047 | | -0.512 | 0.011 |
| Combined | 82 | | -0.205 | 0.121 | 1.092 | | -0.445 | 0.035 |
| ratio = sd(Control)/sd(Treated) | $f=1.441$ | | | | | | | |
| Ho: ratio = 1 | Degrees of freedom = 17, 63 | | | | | | | |
| Ha: ratio < 1 | | Ha: ratio $\neq$ 1 | | | | Ha: ratio > 1 | | |
| $Pr(F<f)=0.8517$ | $2\times Pr(F>f)=0.2965$ | | | | | | $Pr(F>f)=0.1483$ | |

Table S5. Variance Ratio Test: Arousal Baseline (no stimuli) by Treatment Status. Note: as we intended to show that the program had an effect on the variance of the treated, we only considered subjects who were tested in the follow-up survey.

| Group | Obs | | Mean | Std. Err | Std. Dev. | | 95% CI  (lower) | 95% CI  (upper) |
| --- | --- | --- | --- | --- | --- | --- | --- | --- |
| Control | 18 | | -0.004 | 0.270 | 1.147 | | -0.575 | 0.566 |
| Treated | 64 | | -0.219 | 0.145 | 1.160 | | -0.509 | 0.071 |
| Combined | 82 | | -0.172 | 0.127 | 1.153 | | -0.426 | 0.081 |
| ratio = sd(Control)/sd(Treated) | $f=0.978$ | | | | | | | |
| Ho: ratio = 1 | Degrees of freedom = 17, 63 | | | | | | | |
| Ha: ratio < 1 | | Ha: ratio $\neq$ 1 | | | | Ha: ratio > 1 | | |
| $Pr(F<f)=0.5073$ | $2\times Pr(F>f)=0.9854$ | | | | | | $Pr(F>f)=0.4927$ | |

Table S6. Variance Ratio Test: Valence Baseline (no stimuli) by Treatment Status. Note: as we intended to show that the program had an effect on the variance of the treated, we only considered subjects who were tested in the follow-up survey.

| Group | Obs | | Mean | Std. Err | Std. Dev. | | 95% CI  (lower) | 95% CI  (upper) |
| --- | --- | --- | --- | --- | --- | --- | --- | --- |
| Control | 18 | | -0.199 | 0.320 | 1.357 | | -0.874 | 0.476 |
| Treated | 64 | | -0.301 | 0.190 | 1.517 | | -0.680 | 0.078 |
| Combined | 82 | | -0.278 | 0.163 | 1.476 | | -0.603 | 0.046 |
| ratio = sd(Control)/sd(Treated) | $f=0.800$ | | | | | | | |
| Ho: ratio = 1 | Degrees of freedom = 17, 63 | | | | | | | |
| Ha: ratio < 1 | | Ha: ratio $\neq$ 1 | | | | Ha: ratio > 1 | | |
| $Pr(F<f)=0.3138$ | $2\times Pr(F<f)=0.6277$ | | | | | | $Pr(F>f)=0.6862$ | |

Table S7. Variance Ratio Test: Difference Valence Positive and Baseline by Treatment Status. Note: as we intended to show that the program had an effect on the variance of the treated, we only considered subjects who were tested in the follow-up survey.

| Group | Obs | | Mean | Std. Err | Std. Dev. | | 95% CI  (lower) | 95% CI  (upper) |
| --- | --- | --- | --- | --- | --- | --- | --- | --- |
| Control | 18 | | 0.096 | 0.291 | 1.233 | | -0.517 | 0.709 |
| Treated | 64 | | -0.265 | 0.185 | 1.480 | | -0.635 | 0.105 |
| Combined | 82 | | -0.186 | 0.158 | 1.430 | | -0.500 | 0.129 |
| ratio = sd(Control)/sd(Treated) | $f=0.693$ | | | | | | | |
| Ho: ratio = 1 | Degrees of freedom = 17, 63 | | | | | | | |
| Ha: ratio < 1 | | Ha: ratio $\neq$ 1 | | | | Ha: ratio > 1 | | |
| $Pr(F<f)=0.2024$ | $2\times Pr(F<f)=0.4048$ | | | | | | $Pr(F>f)=0.7976$ | |

Table S8. Variance Ratio Test: Difference Valence Negative and Baseline by Treatment Status. Note: as we intended to show that the program had an effect on the variance of the treated, we only considered subjects who were tested in the follow-up survey.

| Group | Obs | Mean | Std. Err | Std. Dev. | | 95% CI  (lower) | 95% CI  (upper) |
| --- | --- | --- | --- | --- | --- | --- | --- |
| Control | 52 | 0.120 | 0.094 | 0.680 | | -0.069 | 0.309 |
| Treated | 88 | 0.040 | 0.108 | 1.017 | | -0.176 | 0.255 |
| Combined | 140 | 0.069 | 0.076 | 0.905 | | -0.082 | 0.220 |
| ratio = sd(Control)/sd(Treated) | $f=1.044$ | | | | | | |
| Ho: ratio = 1 | Degrees of freedom = 51, 87 | | | | | | |
| Ha: ratio < 1 | Ha: ratio $\neq$ 1 | | | | Ha: ratio > 1 | | |
| $Pr(F<f)=0.1683$ | $2\times Pr(F<f)=0.3365$ | | | | | $Pr(F>f)=0.8317$ | |

Table S9. Variance Ratio Test: Arousal Baseline (no stimuli) by PSU Registration Status. Note: as we intended to show that the program had an effect on the variance of the treated, we only considered subjects who were tested in the follow-up survey.

| Group | Obs | Mean | Std. Err | Std. Dev. | | 95% CI  (lower) | 95% CI  (upper) |
| --- | --- | --- | --- | --- | --- | --- | --- |
| Control | 52 | -0.130 | 0.132 | 0.953 | | -0.395 | 0.136 |
| Treated | 88 | 0.016 | 0.099 | 0.933 | | -0.182 | 0.213 |
| Combined | 140 | -0.038 | 0.079 | 0.940 | | -0.195 | 0.119 |
| ratio = sd(Control)/sd(Treated) | $f=1.044$ | | | | | | |
| Ho: ratio = 1 | Degrees of freedom = 51, 87 | | | | | | |
| Ha: ratio < 1 | Ha: ratio $\neq$ 1 | | | | Ha: ratio > 1 | | |
| $Pr(F<f)=0.5766$ | $2\times Pr(F>f)=0.8469$ | | | | | $Pr(F>f)=0.4234$ | |

Table S10. Variance Ratio Test: Valence Baseline (no stimuli) by PSU Registration Status. Note: as we intended to show that the program had an effect on the variance of the treated, we only considered subjects who were tested in the follow-up survey.

|  | Baseline | | | Follow up | | |
| --- | --- | --- | --- | --- | --- | --- |
|  | Treated | Control | Total | Treated | Control | Total |
| Participants | 123 | 164 | 287 | 113 | 92 | 205 |
| Valid EEG records | 85 | 110 | 195 | 68 | 68 | 136 |
| Valid EEG w/relevant info | 76 | 104 | 180 | 64 | 52 | 116 |

Table S11. Attrition. “Valid EEG records” indicates those subjects whose EEG data was readable. “Valid EEG w/relevant info” accounts for those subjects with readable EEG data that was also possible to match with test scores.

|  | Control | Treated | Overall Mean | (1) vs (2) | p-value dif. |
| --- | --- | --- | --- | --- | --- |
| Locus Control Test | -0.058 | 0.172 | 0.039 | -0.357 | 0.130 |
|  | (0.070) | (0.165) | (0.068) | (0.208) |  |
| Grit Test | -0.018 | 0.294 | 0.114 | -0.290 | 0.128 |
|  | (0.151) | (0.129) | (0.076) | (0.168) |  |
| Creativity | 0.040 | 0.010 | 0.027 | 0.273 | 0.401 |
|  | (0.134) | (0.158) | (0.093) | (0.305) |  |
| 13 Forces Creativity | -0.083 | -0.137 | -0.106 | 0.062 | 0.845 |
|  | (0.131) | (0.130) | (0.083) | (0.307) |  |
| Arousal RestState | 0.000 | 0.292 | 0.124 | 0.166 | 0.488 |
|  | (0.109) | (0.068) | (0.107) | (0.227) |  |
| Valence RestState | -0.019 | 0.044 | 0.008 | 0.209 | 0.204 |
|  | (0.039) | (0.059) | (0.040) | (0.149) |  |
| Valence Positive | 0.012 | 0.053 | 0.029 | 0.252 | 0.108 |
|  | (0.072) | (0.085) | (0.070) | (0.137) |  |
| Valence Negative | -0.091 | -0.058 | -0.077 | 0.166 | 0.570 |
|  | (0.060) | (0.045) | (0.033) | (0.279) |  |
| Observations | 104 | 76 | 180 | 180 |  |

Table S12. Experimental Balance at Baseline. Note: standard errors are clustered at school level. Specification includes schools, women, and 12th Grade dummies (*** indicates p<0.01, ** indicates p<0.05, * indicates p<0.1).

|  | Control | Treated | Overall Mean | (1) vs (2) | p-value dif. |
| --- | --- | --- | --- | --- | --- |
| Locus Control Test | -0.096 | 0.250 | 0.046 | -0.421 | 0.287 |
|  | (0.119) | (0.102) | (0.057) | (0.360) |  |
| Grit Test | 0.028 | 0.319 | 0.147 | -0.482 | 0.126 |
|  | (0.130) | (0.091) | (0.072) | (0.271) |  |
| Creativity | 0.164 | 0.043 | 0.115 | 0.659 | 0.176 |
|  | (0.166) | (0.165) | (0.111) | (0.430) |  |
| 13 Forces Creativity | -0.004 | -0.099 | -0.043 | 0.329 | 0.425 |
|  | (0.138) | (0.153) | (0.094) | (0.384) |  |
| Arousal RestState | -0.085 | 0.392 | 0.111 | 0.111 | 0.663 |
|  | (0.131) | (0.095) | (0.144) | (0.242) |  |
| Valence RestState | 0.084 | -0.003 | 0.048 | 0.255 | 0.492 |
|  | (0.056) | (0.118) | (0.069) | (0.348) |  |
| Valence Positive | 0.134 | -0.133 | 0.025 | 0.640 | 0.070(*) |
|  | (0.094) | (0.047) | (0.068) | (0.291) |  |
| Valence Negative | 0.012 | -0.121 | -0.043 | 0.411 | 0.416 |
|  | (0.056) | (0.112) | (0.052) | (0.470) |  |
| Observations | 66 | 46 | 112 | 112 |  |

Table S13. Balance of Attrition (missed subjects compared at baseline). Robust standard errors in parentheses (*** indicates p<0.01, ** indicates p<0.05, * indicates p<0.1).

|  | Control | Treated | Overall Mean | (1) vs (2) | p-value dif. |
| --- | --- | --- | --- | --- | --- |
| Locus Control Test | 0.075 | 0.080 | 0.079 | 0.428 | 0.665 |
|  | (0.379) | (0.106) | (0.129) | (0.947) |  |
| Grit Test | 0.026 | 0.291 | 0.214 | 0.057 | 0.949 |
|  | (0.260) | (0.060) | (0.070) | (0.854) |  |
| Creativity | -0.406 | -0.021 | -0.133 | -1.078 | 0.025 |
|  | (0.303) | (0.136) | (0.135) | (0.378) |  |
| 13 Forces Creativity | -0.099 | 0.196 | 0.110 | -0.793 | 0.244 |
|  | (0.358) | (0.144) | (0.148) | (0.624) |  |
| Raven Fluid Intelligence | -0.407 | -0.087 | -0.180 | 0.338 | 0.704 |
|  | (0.173) | (0.113) | (0.098) | (0.855) |  |
| Arousal RestState | 0.171 | -0.370 | -0.212 | 0.290 | 0.676 |
|  | (0.129) | (0.134) | (0.096) | (0.665) |  |
| Valence RestState | 0.304 | -0.394 | -0.191 | 0.046 | 0.957 |
|  | (0.174) | (0.058) | (0.174) | (0.825) |  |
| Valence Positive | 0.195 | -0.388 | -0.218 | 1.284 | 0.403 |
|  | (0.417) | (0.059) | (0.168) | (1.443) |  |
| Valence Negative | 0.142 | -0.520 | -0.327 | 0.157 | 0.856 |
|  | (0.139) | (0.194) | (0.214) | (0.834) |  |
| Observations | 104 | 34 | 48 | 48 |  |

Table S14. Balance of Attrition (new subjects compared at follow-up). Robust standard errors in parentheses (*** indicates p<0.01, ** indicates p<0.05, * indicates p<0.1).

**SI References**

1 Gross, J. J. & John, O. P. Individual differences in two emotion regulation processes: implications for affect, relationships, and well-being. *Journal of personality and social psychology* **85**, 348 (2003).

2 Lerner, J. S., Li, Y. & Weber, E. U. The financial costs of sadness. *Psychological science* **24**, 72-79 (2013).

3 Ochsner, K. N., Bunge, S. A., Gross, J. J. & Gabrieli, J. D. Rethinking feelings: an FMRI study of the cognitive regulation of emotion. *Journal of cognitive neuroscience* **14**, 1215-1229 (2002).

4 Jamieson, J. P., Nock, M. K. & Mendes, W. B. Mind over matter: reappraising arousal improves cardiovascular and cognitive responses to stress. *Journal of experimental psychology: General* **141**, 417 (2012).

5 Sokol-Hessner, P., Camerer, C. F. & Phelps, E. A. Emotion regulation reduces loss aversion and decreases amygdala responses to losses. *Social cognitive and affective neuroscience* **8**, 341-350 (2013).

6 Lerner, J. S., Li, Y., Valdesolo, P. & Kassam, K. S. Emotion and decision making. *Annual review of psychology* **66**, 799-823 (2015).

7 Troy, A. S. & Mauss, I. B. Resilience in the face of stress: Emotion regulation as a protective factor. *Resilience and mental health: Challenges across the lifespan* **1**, 30-44 (2011).

8 Casey, B. J., Getz, S. & Galvan, A. The adolescent brain. *Developmental review* **28**, 62-77 (2008).

9 Hughes, D. M., Yates, M. J., Morton, E. E. & Smillie, L. D. Asymmetric frontal cortical activity predicts effort expenditure for reward. *Social cognitive and affective neuroscience* **10**, 1015-1019 (2015).

10 Ramirez, R., Planas, J., Escude, N., Mercade, J. & Farriols, C. EEG-based analysis of the emotional effect of music therapy on palliative care cancer patients. *Frontiers in psychology* **9**, 254 (2018).

11 Zhao, G. *et al.* Asymmetric hemisphere activation in tenderness: evidence from EEG signals. *Scientific reports* **8**, 1-9 (2018).

12 Daly, I. *et al.* Electroencephalography reflects the activity of sub-cortical brain regions during approach-withdrawal behaviour while listening to music. *Scientific reports* **9**, 1-22 (2019).

13 Gartstein, M. A., Hancock, G. R., Potapova, N. V., Calkins, S. D. & Bell, M. A. Modeling development of frontal electroencephalogram (EEG) asymmetry: Sex differences and links with temperament. *Developmental science* **23**, e12891 (2020).

14 Decety, J., Meidenbauer, K. L. & Cowell, J. M. The development of cognitive empathy and concern in preschool children: A behavioral neuroscience investigation. *Developmental Science* **21**, e12570 (2018).

15 Harmon-Jones, E. Trait anger predicts relative left frontal cortical activation to anger-inducing stimuli. *International Journal of Psychophysiology* **66**, 154-160 (2007).

16 Lin, Y.-P. *et al.* EEG-based emotion recognition in music listening. *IEEE Transactions on Biomedical Engineering* **57**, 1798-1806 (2010).

17 Ramirez, R. & Vamvakousis, Z. in *International Conference on Brain Informatics.* 175-184 (Springer).

18 Rybak, M., Crayton, J. W., Young, I. J., Herba, E. & Konopka, L. M. Frontal alpha power asymmetry in aggressive children and adolescents with mood and disruptive behavior disorders. *Clinical EEG and neuroscience* **37**, 16-24 (2006).

19 O'Neil, H. F., Perez, R. S. & Baker, E. L. *Teaching and measuring cognitive readiness*. (Springer, 2014).

20 West, M. R. *et al.* Promise and paradox: Measuring students’ non-cognitive skills and the impact of schooling. *Educational Evaluation and Policy Analysis* **38**, 148-170 (2016).

21 Kautz, T., Heckman, J. J., Diris, R., Ter Weel, B. & Borghans, L. Fostering and measuring skills: Improving cognitive and non-cognitive skills to promote lifetime success. (2014).

22 Plucker, J. A. & Makel, M. C. Assessment of Creativity. *The Cambridge Handbook of Creativity*, 48 (2010).

23 Querengässer, J. & Schindler, S. Sad but true?-How induced emotional states differentially bias self-rated Big Five personality traits. *BMC Psychology* **2**, 1-8 (2014).

24 Almlund, M., Duckworth, A. L., Heckman, J. & Kautz, T. in *Handbook of the Economics of Education* Vol. 4 1-181 (Elsevier, 2011).

25 Heckman, J. J. & Kautz, T. Hard evidence on soft skills. *Labour economics* **19**, 451-464 (2012).

26 Egana-delSol, P. (Columbia University Academic Commons, 2016).

27 Damasio, A. R. Descartes’ error: Emotion, rationality and the human brain. *New York: Putnam* **352** (1994).

28 Salzman, C. D. & Fusi, S. Emotion, cognition, and mental state representation in amygdala and prefrontal cortex. *Annual review of neuroscience* **33**, 173-202 (2010).

29 Lakoff, G. *The political mind: why you can't understand 21st-century politics with an 18th-century brain*. (Penguin, 2008).

30 Fuster, J. M. *The neuroscience of freedom and creativity: Our predictive brain*. (Cambridge University Press, 2013).

31 Weber, E. U. & Johnson, E. J. Mindful judgment and decision making. *Annual review of psychology* **60**, 53-85 (2009).

32 Lempert, K. M. & Phelps, E. A. Neuroeconomics of emotion and decision making. *Neuroeconomics*, 219-236 (2014).

33 Loewenstein, G. Emotions in economic theory and economic behavior. *American economic review* **90**, 426-432 (2000).

34 Durlak, J. A., Weissberg, R. P., Dymnicki, A. B., Taylor, R. D. & Schellinger, K. B. The impact of enhancing students’ social and emotional learning: A meta‐analysis of school‐based universal interventions. *Child development* **82**, 405-432 (2011).

35 Barnett, W. S. Preschool education and its lasting effects: Research and policy implications. (2008).

36 Gertler, P. *et al.* Labor market returns to an early childhood stimulation intervention in Jamaica. *Science* **344**, 998-1001 (2014).

37 Deming, D. J. The growing importance of social skills in the labor market. *The Quarterly Journal of Economics* **132**, 1593-1640 (2017).

38 Card, D., Ibarrarán, P., Regalia, F., Rosas-Shady, D. & Soares, Y. The labor market impacts of youth training in the Dominican Republic. *Journal of Labor Economics* **29**, 267-300 (2011).

39 Ibarraran, P., Ripani, L., Taboada, B., Villa, J. M. & Garcia, B. Life skills, employability and training for disadvantaged youth: Evidence from a randomized evaluation design. *IZA Journal of Labor & Development* **3**, 1-24 (2014).

40 Calero, C., Diez, V. G., Soares, Y. S., Kluve, J. & Corseuil, C. H. Can arts-based interventions enhance labor market outcomes among youth? Evidence from a randomized trial in Rio de Janeiro. *Labour Economics* **45**, 131-142 (2017).

41 Heller, S. B. *et al.* Thinking, fast and slow? Some field experiments to reduce crime and dropout in Chicago. *The Quarterly Journal of Economics* **132**, 1-54 (2017).

42 Oosterbeek, H., Van Praag, M. & Ijsselstein, A. The impact of entrepreneurship education on entrepreneurship skills and motivation. *European economic review* **54**, 442-454 (2010).

43 Marvel, M. R., Davis, J. L. & Sproul, C. R. Human capital and entrepreneurship research: A critical review and future directions. *Entrepreneurship Theory and Practice* **40**, 599-626 (2016).

44 Kuratko, D. F. *Entrepreneurship: Theory, process, and practice*. (Cengage learning, 2016).

45 Campos, F. *et al.* Teaching personal initiative beats traditional training in boosting small business in West Africa. *Science* **357**, 1287-1290 (2017).

46 Haushofer, J. & Fehr, E. On the psychology of poverty. *science* **344**, 862-867 (2014).

47 Nabi, G., Liñán, F., Fayolle, A., Krueger, N. & Walmsley, A. The impact of entrepreneurship education in higher education: A systematic review and research agenda. *Academy of Management Learning & Education* **16**, 277-299 (2017).

48 Martin, B. C., McNally, J. J. & Kay, M. J. Examining the formation of human capital in entrepreneurship: A meta-analysis of entrepreneurship education outcomes. *Journal of business venturing* **28**, 211-224 (2013).

49 Lerner, J. & Malmendier, U. With a little help from my (random) friends: Success and failure in post-business school entrepreneurship. *The Review of Financial Studies* **26**, 2411-2452 (2013).

50 Astebro, T., Herz, H., Nanda, R. & Weber, R. A. Seeking the roots of entrepreneurship: Insights from behavioral economics. *Journal of Economic Perspectives* **28**, 49-70 (2014).

51 Heckman, J. J., Stixrud, J. & Urzua, S. The effects of cognitive and noncognitive abilities on labor market outcomes and social behavior. *Journal of Labor economics* **24**, 411-482 (2006).

52 Hirschi, T. & Gottfredson, M. Commentary: Testing the general theory of crime. *Journal of research in crime and delinquency* **30**, 47-54 (1993).

53 Jackson, C. K. Non-cognitive ability, test scores, and teacher quality: Evidence from 9th grade teachers in North Carolina. (National Bureau of Economic Research, 2012).

54 Pratt, T. C. & Cullen, F. T. The empirical status of Gottfredson and Hirschi's general theory of crime: A meta‐analysis. *Criminology* **38**, 931-964 (2000).

55 Duckworth, A. L., Peterson, C., Matthews, M. D. & Kelly, D. R. Grit: perseverance and passion for long-term goals. *Journal of personality and social psychology* **92**, 1087 (2007).

56 Watson, D., Clark, L. A. & Tellegen, A. Development and validation of brief measures of positive and negative affect: the PANAS scales. *Journal of personality and social psychology* **54**, 1063 (1988).

57 Takahashi, H. *et al.* Brain activation associated with evaluative processes of guilt and embarrassment: an fMRI study. *Neuroimage* **23**, 967-974 (2004).

58 Partala, T., Jokiniemi, M. & Surakka, V. in *Proceedings of the 2000 symposium on Eye tracking research & applications.* 123-129.

59 Brown, L., Grundlehner, B. & Penders, J. in *2011 Annual International Conference of the IEEE Engineering in Medicine and Biology Society.* 2188-2191 (IEEE).

60 Verma, G. K. & Tiwary, U. S. Multimodal fusion framework: A multiresolution approach for emotion classification and recognition from physiological signals. *NeuroImage* **102**, 162-172 (2014).

61 Bos, D. O. EEG-based emotion recognition. *The influence of visual and auditory stimuli* **56**, 1-17 (2006).

62 Petrantonakis, P. C. & Hadjileontiadis, L. J. Emotion recognition from EEG using higher order crossings. *IEEE Transactions on information Technology in Biomedicine* **14**, 186-197 (2009).

63 Yoon, H. J. & Chung, S. Y. EEG-based emotion estimation using Bayesian weighted-log-posterior function and perceptron convergence algorithm. *Computers in biology and medicine* **43**, 2230-2237 (2013).

64 Korjus, K. *et al.* Personality cannot be predicted from the power of resting state EEG. *Frontiers in human neuroscience* **9**, 63 (2015).

65 John, O. P. & Srivastava, S. The Big Five trait taxonomy: History, measurement, and theoretical perspectives. *Handbook of personality: Theory and research* **2**, 102-138 (1999).

66 Kim, M.-K., Kim, M., Oh, E. & Kim, S.-P. A review on the computational methods for emotional state estimation from the human EEG. *Computational and mathematical methods in medicine* **2013** (2013).

67 Choppin, A. EEG-based human interface for disabled individuals: Emotion expression with neural networks. *Unpublished master’s thesis* (2000).

68 Martinez-Leon, J.-A., Cano-Izquierdo, J.-M. & Ibarrola, J. Are low cost Brain Computer Interface headsets ready for motor imagery applications? *Expert Systems with Applications* **49**, 136-144 (2016).

69 Friedman, D., Shapira, S., Jacobson, L. & Gruberger, M. in *2015 International Conference on Affective Computing and Intelligent Interaction (ACII).* 930-937 (IEEE).

70 Delorme, A. & Makeig, S. EEGLAB: an open source toolbox for analysis of single-trial EEG dynamics including independent component analysis. *Journal of neuroscience methods* **134**, 9-21 (2004).

71 Castellanos, N. P. & Makarov, V. A. Recovering EEG brain signals: Artifact suppression with wavelet enhanced independent component analysis. *Journal of neuroscience methods* **158**, 300-312 (2006).

72 Mitra, P. *Observed brain dynamics*. (Oxford University Press, 2007).

73 Davidson, R. J., Ekman, P., Saron, C. D., Senulis, J. A. & Friesen, W. V. Approach-withdrawal and cerebral asymmetry: emotional expression and brain physiology: I. *Journal of personality and social psychology* **58**, 330 (1990).

74 Aspiras, T. H. & Asari, V. K. in *2011 8th International Conference on Information, Communications & Signal Processing.* 1-5 (IEEE).

75 Bear, M., Connors, B. & Paradiso, M. A. *Neuroscience: Exploring the Brain, Enhanced Edition: Exploring the Brain*. (Jones & Bartlett Learning, 2020).

76 Moretti, D. V. *et al.* Individual analysis of EEG frequency and band power in mild Alzheimer's disease. *Clinical Neurophysiology* **115**, 299-308 (2004).

77 Klimesch, W. EEG alpha and theta oscillations reflect cognitive and memory performance: a review and analysis. *Brain research reviews* **29**, 169-195 (1999).

78 Klimesch, W. Memory processes, brain oscillations and EEG synchronization. *International journal of psychophysiology* **24**, 61-100 (1996).

79 Harmon-Jones, E., Gable, P. A. & Peterson, C. K. The role of asymmetric frontal cortical activity in emotion-related phenomena: A review and update. *Biological psychology* **84**, 451-462 (2010).

80 Keenan, S. A. Normal human sleep. *Respiratory Care Clinics of North America* **5**, 319-331, vii (1999).

81 Knyazev, G. G. Motivation, emotion, and their inhibitory control mirrored in brain oscillations. *Neuroscience & Biobehavioral Reviews* **31**, 377-395 (2007).

82 Schaffer, C. E., Davidson, R. J. & Saron, C. Frontal and parietal electroencephalogram asymmetry in depressed and nondepressed subjects. *Biological psychiatry* (1983).

83 Kassam, K. S., Markey, A. R., Cherkassky, V. L., Loewenstein, G. & Just, M. A. Identifying emotions on the basis of neural activation. *PloS one* **8**, e66032 (2013).

84 Renard, Y. *et al.* Openvibe: An open-source software platform to design, test, and use brain–computer interfaces in real and virtual environments. *Presence* **19**, 35-53 (2010).

85 Bokil, H., Andrews, P., Kulkarni, J. E., Mehta, S. & Mitra, P. P. Chronux: a platform for analyzing neural signals. *Journal of neuroscience methods* **192**, 146-151 (2010).

86 Alarcao, S. M. & Fonseca, M. J. Emotions recognition using EEG signals: A survey. *IEEE Transactions on Affective Computing* **10**, 374-393 (2017).

87 Williams, N. S., McArthur, G. M. & Badcock, N. A. 10 years of EPOC: A scoping review of Emotiv’s portable EEG device. *BioRxiv* (2020).

88 Mladenov, T., Kim, K. & Nooshabadi, S. in *2012 IEEE 16th International Symposium on Consumer Electronics.* 1-4 (IEEE).

89 Taylor, G. S. & Schmidt, C. in *Proceedings of the Human Factors and Ergonomics Society Annual Meeting.* 193-197 (SAGE Publications Sage CA: Los Angeles, CA).

90 de Lissa, P., Sörensen, S., Badcock, N., Thie, J. & McArthur, G. Measuring the face-sensitive N170 with a gaming EEG system: a validation study. *Journal of neuroscience methods* **253**, 47-54 (2015).

91 Duvinage, M. *et al.* Performance of the Emotiv Epoc headset for P300-based applications. *Biomedical engineering online* **12**, 1-15 (2013).

92 Badcock, N. A. *et al.* Validation of the Emotiv EPOC® EEG gaming system for measuring research quality auditory ERPs. *PeerJ* **1**, e38 (2013).

93 Rotter, J. B. Generalized expectancies for internal versus external control of reinforcement. *Psychological monographs: General and applied* **80**, 1 (1966).

94 Lee, D. S. Training, wages, and sample selection: Estimating sharp bounds on treatment effects. *The Review of Economic Studies* **76**, 1071-1102 (2009).

95 Haushofer, J. & Shapiro, J. The short-term impact of unconditional cash transfers to the poor: experimental evidence from Kenya. *The Quarterly Journal of Economics* **131**, 1973-2042 (2016).

96 Jones, D., Molitor, D. & Reif, J. What do workplace wellness programs do? Evidence from the Illinois workplace wellness study. *The Quarterly Journal of Economics* **134**, 1747-1791 (2019).

97 Cameron, A. C. & Trivedi, P. K. *Microeconometrics: methods and applications*. (Cambridge university press, 2005).

98 Attanasio, O., Cattan, S., Fitzsimons, E., Meghir, C. & Rubio-Codina, M. Estimating the production function for human capital: results from a randomized controlled trial in Colombia. *American Economic Review* **110**, 48-85 (2020).

99 Card, D., Kluve, J. & Weber, A. What works? A meta analysis of recent active labor market program evaluations. *Journal of the European Economic Association* **16**, 894-931 (2018).

100 Anderson, M. L. Multiple inference and gender differences in the effects of early intervention: A reevaluation of the Abecedarian, Perry Preschool, and Early Training Projects. *Journal of the American statistical Association* **103**, 1481-1495 (2008).

101 Kling, J. R., Liebman, J. B. & Katz, L. F. Experimental analysis of neighborhood effects. *Econometrica* **75**, 83-119 (2007).

102 Casey, K., Glennerster, R. & Miguel, E. Reshaping institutions: Evidence on aid impacts using a preanalysis plan. *The Quarterly Journal of Economics* **127**, 1755-1812 (2012).

103 Russell, J. A. A circumplex model of affect. *Journal of personality and social psychology* **39**, 1161 (1980).

104 Yu, L.-C. *et al.* in *Proceedings of the 2016 Conference of the North American Chapter of the Association for Computational Linguistics: Human Language Technologies.* 540-545.

1. We refer to dual-emotion solution as the presence of a counteracting emotional state. [↑](#footnote-ref-1)
2. We are not arguing that it is misleading to generalize results obtained from self-reported psychometric tests; we only argue that, in the context of an intervention that targets some emotional dimension, it is empirically impossible to disentangle from those tests the impact of any emotional bias linked to the actual change due the program or other sources of bias, such as reference bias or test anxiety. [↑](#footnote-ref-2)
3. Reference bias can affect self-reporting on socio-emotional skills measures. [↑](#footnote-ref-3)
4. As mentioned, we also explored strategies suited to exempt self-reported psychometric tests from emotional bias using the methods drawn from affective neuroscience found in Egana-delSol's work (26). [↑](#footnote-ref-4)
5. PANAS asks subjects to rate, on a scale from 1 to 10, how intensely they feel each of seven positive and nine negative effects. The positive effects are amusement, arousal, contentment, happiness, interest, relief, and surprise; the negative effects are anger, confusion, contempt, disgust, embarrassment, fear, pain, sadness, and tension. In addition, subjects were asked whether the film clip made them happier, sadder, or neither; and whether the film clip put them in a better mood, worse mood, or neither. [↑](#footnote-ref-5)
6. As mentioned, we explored strategies suited to correct self-reported psychometric tests using neurophysiological methods in Egana-delSol's work (26). We argue that valence has a positive correlation: positive/approach emotions (pleased, happy) imply overestimated self-reports, while negative/withdrawal emotions (unhappy, sad) would cause an underestimation of the self-perceptions of certain socio-emotional skills. However, for arousal, the effect is ambiguous (31). The empirical findings support this hypothesis. [↑](#footnote-ref-6)
7. For instance, as mentioned above, Ekman et al. suggested the universality of six facial expressions based on Darwinian theory. However, Partala et al. (58) argued that facial expressions can be easily simulated by subjects. Moreover, arousal effects on behavior, self-reporting and decision-making are ambiguous. [↑](#footnote-ref-7)
8. Electroencephalogram (EEG) devices measure the voltage change that occurs when a neuron fires. When a positive change in the voltage crosses a certain threshold, an action potential is triggered. Indeed, the voltage goes from a resting potential of about -60mV to +20mV. This electrical activity from group of neurons is measured in the cortex by the EEG's electrodes. That is to say that an EEG measures the brain’s activity through the voltage changes in groups of neurons that have been fired due an action potential. [↑](#footnote-ref-8)
9. Verma and Tiwary (60) propose a fusion model that encompass other physiological measures in addition to arousal and valence indices. Regardless, the authors estimations indicate that the *Fusion* and *Circumplex* -- arousal-valence -- models have similar accuracy. [↑](#footnote-ref-9)
10. The dominance scale ranges from submissive (or without control) to dominant (or in control, empowered). [↑](#footnote-ref-10)
11. In a lab setting, Ramirez and Vamvakousis (17) use the same low-cost portable EEG device used in the present study. [↑](#footnote-ref-11)
12. Biosemi. For details visit <http://www.biosemi.com/>. [↑](#footnote-ref-12)
13. Emotiv EPOC EEG. For details visit <http://www.emotiv.com/>. [↑](#footnote-ref-13)
14. A MATLAB toolbox developed by Mitra and Bokil (72) to implement those methods is freely available at <http://chronux.org/chronux/>. [↑](#footnote-ref-14)
15. The exact transformation follows Aspiras and Asari (74). In particular, the EEG signals were transformed as follow: $\log{signal}_{j}=10\times{log}_{10} {(signal}_{j})$, where $j$ indicates different conditions (positive, negative, or resting state). [↑](#footnote-ref-15)
16. There is ample data linking changes in these frequency bands to various mental processes such as changes in mental state (76), changes in attention allocated to a task (77), memory processes (78), motivation and emotional processes (81), and different sleep stages (80), among others. Here we focus on arousal and positive valence/approach behavior and negative valence/withdrawal behavior (60, 73, 79). [↑](#footnote-ref-16)
17. Discussion on the validity of estimate valence by comparing hemispherical activation (17, 60, 61, 63, 66, 69, 73, 79). [↑](#footnote-ref-17)
18. The latter problem can be ameliorated by breaking up the observation window into shorter segments and averaging spectral estimates computed over those segments. However, this is typically impossible for neural time-series, which are stationary only over relatively short durations of a few hundred milliseconds (84). [↑](#footnote-ref-18)
19. A MATLAB toolbox for implementing those methods is freely available at [http://chronux.org/chronux](http://chronux.org/chronux%2520) (72). [↑](#footnote-ref-19)
20. For instance, a recent study found high quality and low variance in EEG recordings with the low-cost Emotiv EPOC, as opposed to the professional-grade Biosemi Active II, thereby supporting the use of consumer-grade systems for emotion detection in the lab and field (68). There is more research showing the reliability of Emotiv EPOC (86, 88-92) *(100, 102-106)* [↑](#footnote-ref-20)
21. The full GAPED dataset is freely available at <http://www.affective-sciences.org/researchmaterial> [↑](#footnote-ref-21)
22. For further details on the procedures see Jones et al. (96) [↑](#footnote-ref-22)
23. See Cameron and Trivedi (97) for details on Probit models. [↑](#footnote-ref-23)
24. See Cameron and Trivedi (97) for details on model selection based on information criteria. [↑](#footnote-ref-24)
25. The PSU is a high-stakes test to apply to college similar to the SAT in the U.S. [↑](#footnote-ref-25)
26. In unreported results, we find no impact on PSU scores themselves. [↑](#footnote-ref-26)
27. We also estimated the minimum detectable effects for each dimension based on a power of 80% and a significance of 5%. [↑](#footnote-ref-27)
28. The James-Lange theory construct valence and arousal indices based on alpha and beta waves in the pre-frontal cortex (17, 26, 60, 73, 79) [↑](#footnote-ref-28)
29. In many studies similar to our own, researchers employed the Positive and Negative Affect Schedule (PANAS), a self-reported method to evaluate emotions (see Egana-delSol’s work (26) and the literature referred therein). In fact, besides its shortcomings with regard to self-reporting biases, PANAS would have made salient the relevance of emotions in this experiment, potentially affecting both reaction to stimuli and self-reports on emotional state and other dimensions. Therefore, this instrument was not considered for this study. [↑](#footnote-ref-29)
30. For example, one laptop was having an internal problem and recorded with a high proportion of error for two consecutive days, which generated 13 missing subjects during these days. [↑](#footnote-ref-30)
